# Development and validation of a population-based risk stratification model for severe COVID-19 in the general population

**DOI:** 10.1038/s41598-022-07138-y

**Published:** 2022-02-28

**Authors:** Emili Vela, Gerard Carot-Sans, Montse Clèries, David Monterde, Xènia Acebes, Adrià Comella, Luís García Eroles, Marc Coca, Damià Valero-Bover, Pol Pérez Sust, Jordi Piera-Jiménez

**Affiliations:** 1 Servei Català de la Salut (CatSalut), Barcelona, Spain; 2 Digitalization for the Sustainability of the Healthcare System (DS3), IDIBELL, Barcelona, Spain; 3 Sistemes d’Informació, Institut Català de La Salut, Barcelona, Catalonia Spain; 4 Open Evidence Research Group, Universitat Oberta de Catalunya, Barcelona, Spain

**Keywords:** Health care, Risk factors

## Abstract

The shortage of recently approved vaccines against the severe acute respiratory syndrome coronavirus 2 (SARS-CoV-2) has highlighted the need for evidence-based tools to prioritize healthcare resources for people at higher risk of severe coronavirus disease (COVID-19). Although age has been identified as the most important risk factor (particularly for mortality), the contribution of underlying comorbidities is often assessed using a pre-defined list of chronic conditions. Furthermore, the count of individual risk factors has limited applicability to population-based “stratify-and-shield” strategies. We aimed to develop and validate a COVID-19 risk stratification system that allows allocating individuals of the general population into four mutually-exclusive risk categories based on multivariate models for severe COVID-19, a composite of hospital admission, transfer to intensive care unit (ICU), and mortality among the general population. The model was developed using clinical, hospital, and epidemiological data from all individuals among the entire population of Catalonia (North-East Spain; 7.5 million people) who experienced a COVID-19 event (i.e., hospitalization, ICU admission, or death due to COVID-19) between March 1 and September 15, 2020, and validated using an independent dataset of 218,329 individuals with COVID-19 confirmed by reverse transcription—polymerase chain reaction (RT-PCR), who were infected after developing the model. No exclusion criteria were defined. The final model included age, sex, a summary measure of the comorbidity burden, the socioeconomic status, and the presence of specific diagnoses potentially associated with severe COVID-19. The validation showed high discrimination capacity, with an area under the curve of the receiving operating characteristics of 0.85 (95% CI 0.85–0.85) for hospital admissions, 0.86 (0.86–0.97) for ICU transfers, and 0.96 (0.96–0.96) for deaths. Our results provide clinicians and policymakers with an evidence-based tool for prioritizing COVID-19 healthcare resources in other population groups aside from those with higher exposure to SARS-CoV-2 and frontline workers.

## Introduction

The vaccines against the severe acute respiratory syndrome coronavirus 2 (SARS-CoV-2) have changed the course of the COVID-19 pandemic in many countries worldwide. However, the massive number of doses needed to achieve herd immunity will likely lead to a scarcity of the marketed vaccines. This scenario, which may worsen if long-term immunity is not achieved^1^, will force governments to establish priority criteria for accessing vaccines. This prioritization also applies to other healthcare resources needed for preventive strategies such as screening campaigns, awareness programs, and early administration of specific therapies that are not widely available.

Aside from protecting highly exposed individuals like healthcare workers, the risk of serious illness seems to be the most reasonable criterion to prioritize access to COVID-19 resources based on a “stratify-and-shield” strategy^2^. Various studies have identified age as the most important predictive factor for mortality in COVID-19 hospitalized patients^3,4^. Thus, in the absence of a consensus framework for COVID-19 risk allocation, age at the cut-off of 65 years has been proposed as a criterion for targeting populations for vaccine prioritization^5^. However, an age cut-off as a sole criterion for risk stratification might not accurately define the risk of severe illness^6^ and has raised ethical concerns^7^. To date, various factors—aside from age—have been associated with severe illness^8–10^. Based on these factors, various prediction models for COVID-19 have been proposed^11^. While most of these models are based on data from cohorts of limited size or aimed at estimating risk in specific populations like hospitalized patients, others have used nationwide approaches to develop scores for predicting the risk of complications or severe illness based on baseline health information (i.e., before COVID-19 onset) existing in electronic health records^12–14^. In countries with centralized electronic health records, these types of models may help policymakers in targeting public health and containment campaigns and prioritizing resources (e.g., vaccines, diagnostic tests, and hospital and intensive care unit [ICU] beds) based on the baseline risk of the population. Models presented to date identify the comorbidity burden among a factor contributing to the risk of severe illness; however, multimorbidity is typically measured by the presence (and/or unweighted counts) of diagnoses from a predefined list of chronic conditions. Alternatively, exhaustive measures of multimorbidity based on weighted counts of all chronic conditions (and relevant and recent acute diagnoses) may provide a more accurate perspective of patients’ health risk^15,16^.

Using whole-population data on hospitalizations, ICU transfers, and deaths due to COVID-19 in our area, we aimed to develop and validate a population-based model intended to stratify the general population according to their risk of serious events due to COVID-19. Based on the ideal characteristics of such stratification system suggested elsewhere^6^, we sought a system that was population-based (i.e., all individuals in a community could be assigned to mutually-exclusive groups), accessible (i.e., it must be based on information available and accessible to all healthcare professionals), understandable (i.e., it must be easily explained to policymakers and citizens), discriminatory (i.e., individuals could be allocated in a discrete list of strata), and suitable for local implementation.

## Methods

### Study design, population, and data sources

This was an observational retrospective population-based study of severe COVID-19 risk in Catalonia, a North-East region in Spain with a population of 7.5 million people. The study included two phases, conducted using two different populations. First, owing to the lack of accurate diagnostic information among COVID-19 outpatients during the first wave of the pandemic, we developed a predictive model by considering all patients hospitalized, admitted to ICU, or dying because of COVID-19 among the entire population of Catalonia. The model was developed using data collected between March 1 and September 15, 2020 (development period), which encompassed the first wave of the COVID-19 outbreak in our area and a period between waves. The scarcity of PCR tests during the first wave of the pandemic precluded the testing of all suspected cases of COVID-19. For that reason, in cases collected during the first wave of the development period, we considered the COVID-19 diagnosis according to either molecular criteria (positive result with a PCR or serological test) or clinical/epidemiological (i.e., reported as COVID-19 case in the electronic health records, based on the criteria of the European Centre for Disease Prevention and Control [ECDC]^17^ in force by the time of diagnosis), as officially established in the Aggregated Healthcare Registry for COVID-19 (RSACovid-19, for Catalan *Registres Sanitaris Agregats*). Second, we validated the risk model by investigating the occurrence of hospitalization, ICU admission, and death among individuals diagnosed with COVID-19. All cases collected for the validation period had a positive result in a PCR test. Data for model validation had been collected between September 16 and December 27, 2020 (i.e., the date the first vaccine was administered in Catalonia) (validation period). The database was closed on Mar 3, 2021, thus capturing all hospitalizations, ICU admissions, and deaths occurring up to this date.

Data on potential predictors were retrieved from the Catalan Health Surveillance System (CHSS), which systematically collects data regarding diagnoses, individual income, and resource utilization from both hospital and primary care settings^18^. The hospital and primary care databases are linked through a unique identification number used for public assurance purposes. Diagnoses are introduced in the registry using the codes of the international classification of diseases v10 clinical modification (ICD-9-CM); the smoking status is gathered through the anamnesis. ICD-9-CM codes are listed in the Supplementary file 1.This information originates from the interactions between patients and any healthcare entity or service and is regularly transferred from electronic health records of healthcare providers to the Catalan Health Service (the public insurer in Catalonia), which uses it for billing purposes, among others. The CHSS is updated once yearly. Data on diagnoses and outcomes associated with SARS-CoV-2 infection were retrieved from the epidemiological surveillance system in the SARS-CoV-2 registry (RSACovid-19), which centralizes all data regarding SARS-CoV-2 testing and COVID-19 diagnosis from all healthcare centres in the area^19,20^. The first author had full access to the datasets. According to the sequencing surveillance system, the alpha variant became dominant by August 2020, and remained dominant during the validation period (Figure S1, Supplementary File 1).

All data were handled according to the General Data Protection Regulation 2016/679 on data protection and privacy for all individuals within the European Union and the local regulatory framework regarding data protection. Data from different health administrative databases were linked and de-identified by a team not involved in the study analysis; study investigators only had access to a fully anonymized database. The retrospective use of healthcare data was approved by the Independent Ethics Committee of the IDIAP Jordi Gol (Spain), which waived the need for obtaining informed consent for data utilization. Results are presented in accordance to the Transparent reporting of a multivariable prediction model for individual prognosis or diagnosis (TRIPOD) guidelines. STROBE and RECORD guidelines for observational studies and studies using routinely collected health data were also considered.

### Predictors

We considered all variables stored in the CHSS database, including demographic data (i.e., age and sex), resource utilization (e.g., admission to nursing homes), lifestyle information (e.g., smoking, and alcohol abuse), current and past diagnoses (including psychiatric disorders), and socioeconomic status. The global comorbidity burden (or patient complexity) was stratified using the adjusted morbidity groups (GMA, *Grups de Morbiditat Ajustada*), a population-based tool for health-risk assessment^21–23^. The GMA tool considers the weighted sum of all chronic conditions, the number of systems affected, and acute diagnoses present at the time that may increase patient complexity. Individuals are grouped into four health-risk categories defined using the risk distribution of the entire population: (1) baseline risk (healthy stage, including GMA scores up to the 50th percentile of the total population), (2) low risk, 50th to 80th percentiles, (3) moderate risk, 80th to 95th percentiles, and (4) high risk, above the 95th percentile. Socioeconomic status was stratified according to pharmaceutical co-payment groups, which are based on annual income, as follows: very low (i.e., recipient of rescue aid measures), low (i.e., less than € 18,000), middle (i.e., € 18,000 to € 100,000), and high (i.e., > € 100,000). Owing to the lack of standardized treatments within the model development period, no treatment-related variables were included in the model.

### Outcomes

We analysed three outcomes associated with severe COVID-19: hospital admission, transfer to intensive care unit (ICU), and death. Owing to the shortage of ICU beds during the first wave (March 03 to July 15, 2020), the start of invasive mechanical ventilation was considered an ICU transfer, irrespective of an ICU admission registry. All deaths related to COVID-19 were included, whether they had been hospitalized or not.

### Statistics

The dataset for developing the stratification model included all individuals covered by the public health system in Catalonia (development cohort). Owing to the population-based approach, no formal estimate of the sample size was done. The inclusion in the dataset was event-driven; all factors considered were either clinical conditions or variables required for being registered as a user of the healthcare system (e.g., age, sex, annual income); therefore, there were no missing data in the variables analysed; no imputation for missing data was applied. Categorical variables were summarized as frequency and percentage, whereas continuous variables were represented by the median and interquartile range (IQR, defined as the 25th and 75th percentiles). We used generalized linear models (Poisson regression) to build multivariate models for hospitalizations, ICU transfers, and deaths due to COVID-19, with the contribution of each factor expressed as a risk ratio (RR) and its 95% confidence interval (CI). The models were created using a "stepwise-forward" approach based on the Akaike Information Criterion (AIC), in which a naïve model is sequentially complemented with the most relevant variables, eventually leading to the main effects model^24^. The resulting delta values are provided in Table S1. The variables included in the model are listed in the Supplementary file 1. The model was then refined to yield the final model as follows: owing to its non-linear behaviour, age was introduced into the final model as a continuous variable plus an additional quadratic term; the models also included all significant first-order interactions between selected variables and sex and age. The final models provided individual-level estimates of the probability for each outcome (i.e., hospitalization, transfer to ICU, and death) for the entire population of Catalonia. Results were presented as the main effects model and the full model including all interactions. The accuracy of the three models was assessed using the area under the curve of the receiving operating characteristics (AUC ROC) of the full model. The four risk strata were defined by crosslinking the three categorized probabilities.

The stratification model was validated using an independent dataset of all individuals with a positive reverse transcription—polymerase chain reaction (RT-PCR) result for SARS-CoV-2 infection in a respiratory specimen within the validation period (validation cohort). Observed cases included the sum of all events occurred within the analysed period (i.e., hospitalization, ICU admission, and deaths due to COVID-19), whereas the estimated cases were the sum of individual probabilities provided by the models. The goodness of fit of the model was assessed using the AUC ROC and the corresponding 95% confidence interval for each outcome. The model was not updated after validation. The significance threshold was set at an alpha error of 0.05; all analyses were performed using R statistical software, version R-4.0.0^25^.

### Ethics approval

The study protocol was approved by the Independent Ethics Committee of the IDIAP Jordi Gol (Spain), which waived the need for written informed consent (21/043-PCV).

### Reporting guidelines

Results are presented in accordance to the Trans parent reporting of a multivariable prediction model for individual prognosis or diagnosis (TRIPOD) guidelines.

## Results

### Model development and specification

**Figure 1:**
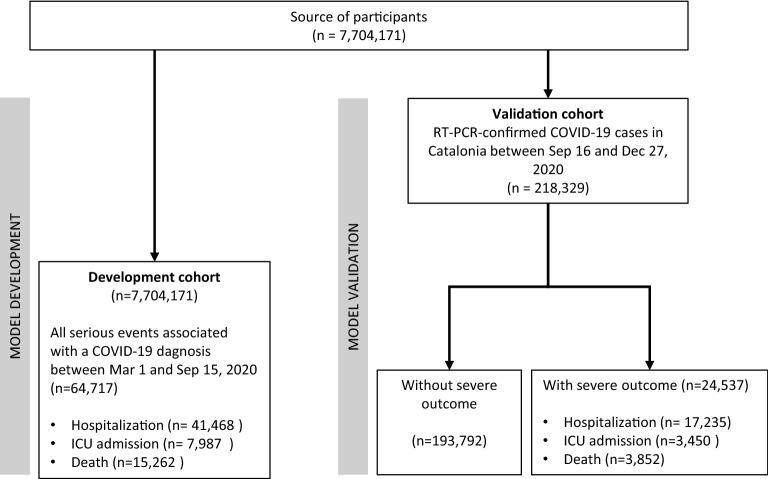
Flow-chart of individual inclusion for the development and validation cohorts. *RT-PCR* reverse transcription–polymerase chain reaction.

Figure 1 shows the flow-chart of individual inclusion in the development and validation data sets. The main characteristics of individuals included in each dataset are shown in Table 1. The generalized linear models for model development were built from data on 41,468 hospitalizations, 7987 ICU transfers, and 15,262 deaths (all of them associated with COVID-19), which occurred during the first six months of the outbreak in Catalonia (development period). Overall, 77.9% of individuals of the development dataset were RT-PCR-confirmed; the percentage of individuals identified using other diagnostic criteria are shown in Table S2. These events corresponded to a population rate (per 1000 persons per year) of 9.9 hospitalizations, 1.9 ICU admissions, and 3.7 deaths. The resulting main effects models for hospitalization, ICU transfer, and mortality are summarized in Figure S2, whereas the full models used for stratification are described in Tables S3 to S5 of the Supplementary file 1.

**Table 1:** Main characteristics of individuals included in the development and validation cohorts.

	Development (*n* = 7,704,171)	Validation (*n* = 218,329)
Age (years), median [IQR]	43.0 [24.0;59.0]	41.0 [22.0;57.0]
**Socioeconomic status**^**a**^		
High	90,521 (1.17%)	2177 (1.00%)
Moderate	2,725,258 (35.4%)	71,312 (32.7%)
Low	4,620,504 (60.0%)	135,248 (61.9%)
Very low	267,888 (3.48%)	9592 (4.39%)
**Health risk (GMA level)**^**b**^		
Basal risk	3,863,727 (50.2%)	103,384 (47.4%)
Low risk	2,334,573 (30.3%)	69,402 (31.8%)
Moderate risk	1,159,138 (15.0%)	32,333 (14.8%)
High risk	346,733 (4.50%)	13,210 (6.05%)
Smoker	1,315,588 (17.1%)	29,767 (13.6%)
Nursing home resident	71,158 (0.92%)	5579 (2.56%)
**Relevant clinical conditions**^**c**^		
Diabetes mellitus	590,341 (7.66%)	18,588 (8.51%)
Heart failure	197,798 (2.57%)	7414 (3.40%)
COPD	362,491 (4.71%)	10,635 (4.87%)
Hypertension	1,552,488 (20.2%)	43,796 (20.1%)
AIDS-HIV	28,545 (0.37%)	687 (0.31%)
Ischemic heart disease	235,640 (3.06%)	7201 (3.30%)
Stroke	245,723 (3.19%)	8536 (3.91%)
Chronic kidney disease	327,639 (4.25%)	11,121 (5.09%)
Dementia	85,833 (1.11%)	4812 (2.20%)
Obesity	1,250,330 (16.2%)	41,249 (18.9%)
Hyperlipidaemia	1,298,582 (16.9%)	35,913 (16.4%)
Active neoplasm	281,631 (3.66%)	7616 (3.49%)
Severe intellectual disability	7966 (0.10%)	432 (0.20%)
Psychiatric chronic disease	452,995 (5.88%)	12,558 (5.75%)

^a^Grouped according to the annual income as follows: very low (i.e., recipient of rescue aid measures), low (i.e., less than € 18,000), middle (i.e., € 18,000 to € 100,000), and high (i.e., > € 100,000). ^b^ Grouped according to the adjusted morbidity groups (GMA) index, based on the distribution of the entire population into the following groups: baseline risk (healthy stage, including GMA scores up to the 50th percentile of the total population), low risk (50th to 80th percentiles), moderate risk (80th to 95th percentiles), and high risk (above the 95th percentile). ^c^ Categories are not mutually exclusive.

*AIDS-HIV* acquired immunodeficiency syndrome by human immunodeficiency virus. *COPD* Chronic obstructive pulmonary disease. *IQR* interquartile range, defined as the 25th and 75th percentiles.

The four mutually exclusive groups of low, moderate, high, and very high risk were defined based on the crosslinking probabilities of the three outcomes. First, the distribution of probabilities resulting from the full models led to three risk groups (low, moderate, and high) for each outcome, defined according to the following percentile thresholds that maximized group separation in our population: percentiles 50th (probability 2.64 × 10^–3^) and 80th (probability 7.04 × 10^–3^) for hospitalization, percentiles 50th (probability 0.27 × 10^–3^) and 85th (probability 1.24 × 10^–3^) for ICU admission, and percentiles 77th (probability 0.58 × 10^–3^) and 92th (probability 3.57 × 10^–3^) for death. This risk groups for each outcome were then combined to obtain the four risk groups of the model. The *very-high risk group* included all individuals with high risk for the outcome death. The *high-risk group* included all individuals with either moderate risk for death or low risk for death but high risk for hospitalization and high risk of ICU admission. The *moderate-risk group* included all individuals with low risk for death but one of the following situations: (1) high risk for hospitalization and low-to-moderate risk for ICU admission, (2) moderate risk for hospitalization and moderate-to-high risk for ICU admission, or (3) low risk for hospitalization and high risk for ICU admission. The *low-risk group* included individuals meeting the remaining risk combinations for the three outcomes: (1) low risk for death, moderate risk for hospitalization, and low risk for ICU admission, or (2) low risk for death, low risk for hospitalization and low-to-moderate risk for ICU admission.

**Figure 2:**
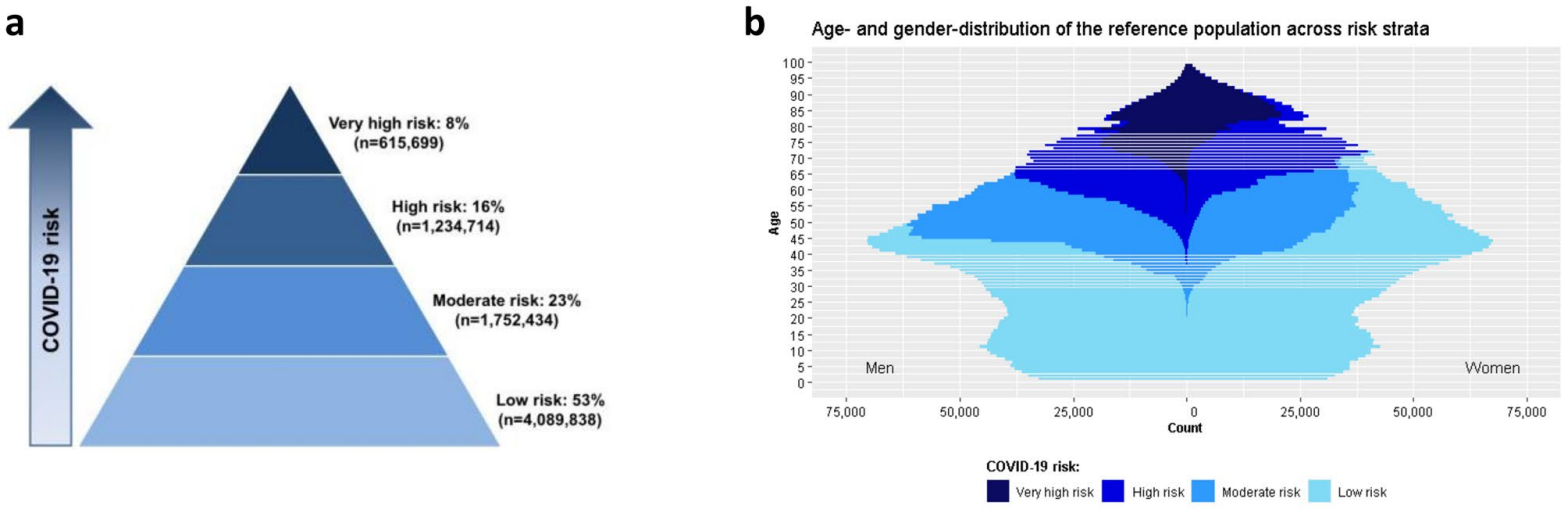
Distribution of the reference population (i.e., Catalonia, 7,697,069 inhabitants) across risk groups. (**a**) percentage of individuals allocated in each risk group. (**b**) age distribution across risk groups.

Figure 2 shows the proportion of individuals allocated to each group and the age distribution across risk groups for the reference population. Model calibration showed low discrepancy between observed and expected cases during the development period (Figure S4). ROC AUC (95% CI) for hospitalizations, ICU transfers, and deaths were 0.82 (0.814–0.82), 0.83 (0.83–0.84), and 0.96 (0.96–0.96), respectively.

Figure S3 summarizes the demographic and clinical profile of individuals allocated in each risk group. Briefly, the low-risk group had 55% women, with a median age of 26 years (IQR 13–38) and a very low prevalence of comorbidities; this group covered the healthy population. Individuals in the moderate-risk group were mostly men (66%) with a median age of 50 years (IQR 45–55) and a low comorbidity burden. A remarkable percentage of individuals diagnosed with AIDS (43.4%) or severe psychiatric disorders (30.7%) among the overall population fell into this group. The high-risk group had 51% of women with median age of 67 years (IQR 62–73). This group typically included middle-aged adults with cardiovascular risk factors; 54.6% of all individuals with hypertension, 43.5% of those with hyperlipidemia, and 35.6% of those with obesity fell into this group. The very high-risk group had 45% women with a median age of 82 years (IQR 76–87). This group included almost all people institutionalized in a nursing home (91.6%), diagnosed with dementia (89.7%), and receiving domiciliary care (87.6%). A remarkable percentage of individuals with kidney failure (64.8%), heart failure (69.5%), ischemic heart disease (53.8%), and stroke (51.6%) among the overall population also fell into the very high-risk group.

### Validation of the stratification model

**Figure 3:**
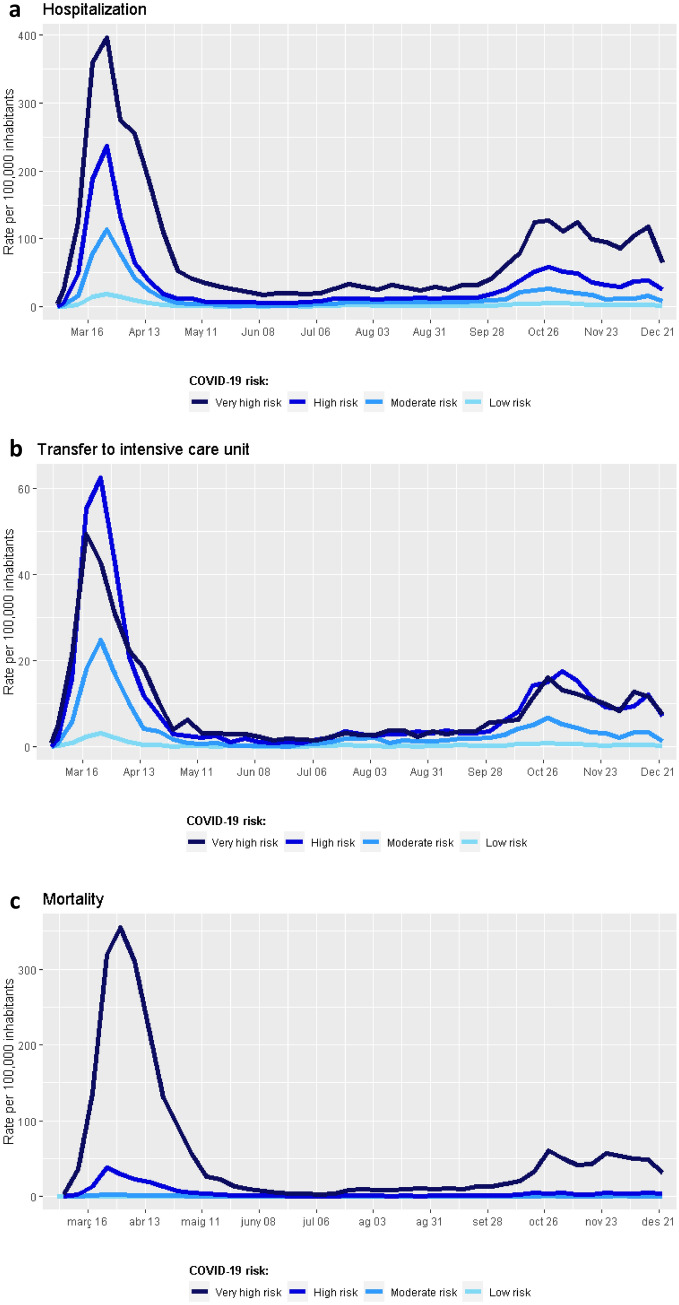
Longitudinal analysis of outcome rate within the first 10 months of the COVID-19 pandemic in Catalonia. Results are presented as the incidence rate at the population level and stratified according to COVID-19 risk group. (**a**) Hospital admissions due to COVID-19. (**b**) Transfer to an intensive care unit (ICU) due to COVID-19. (**c**) Death due to COVID-19.

The weekly rate of hospitalizations among the general population increased with risk groups during the entire period, being the differences between groups more pervasive during waves (Fig. 3a). The other two outcomes also displayed an increasing trend across risk groups. However, the rate of ICU transfers was similar in the very high- and high-risk groups during the second wave, and mortality clearly stood out among the individuals of the very high-risk group during the two waves (Fig. 3b and c, respectively).

**Figure 4:**
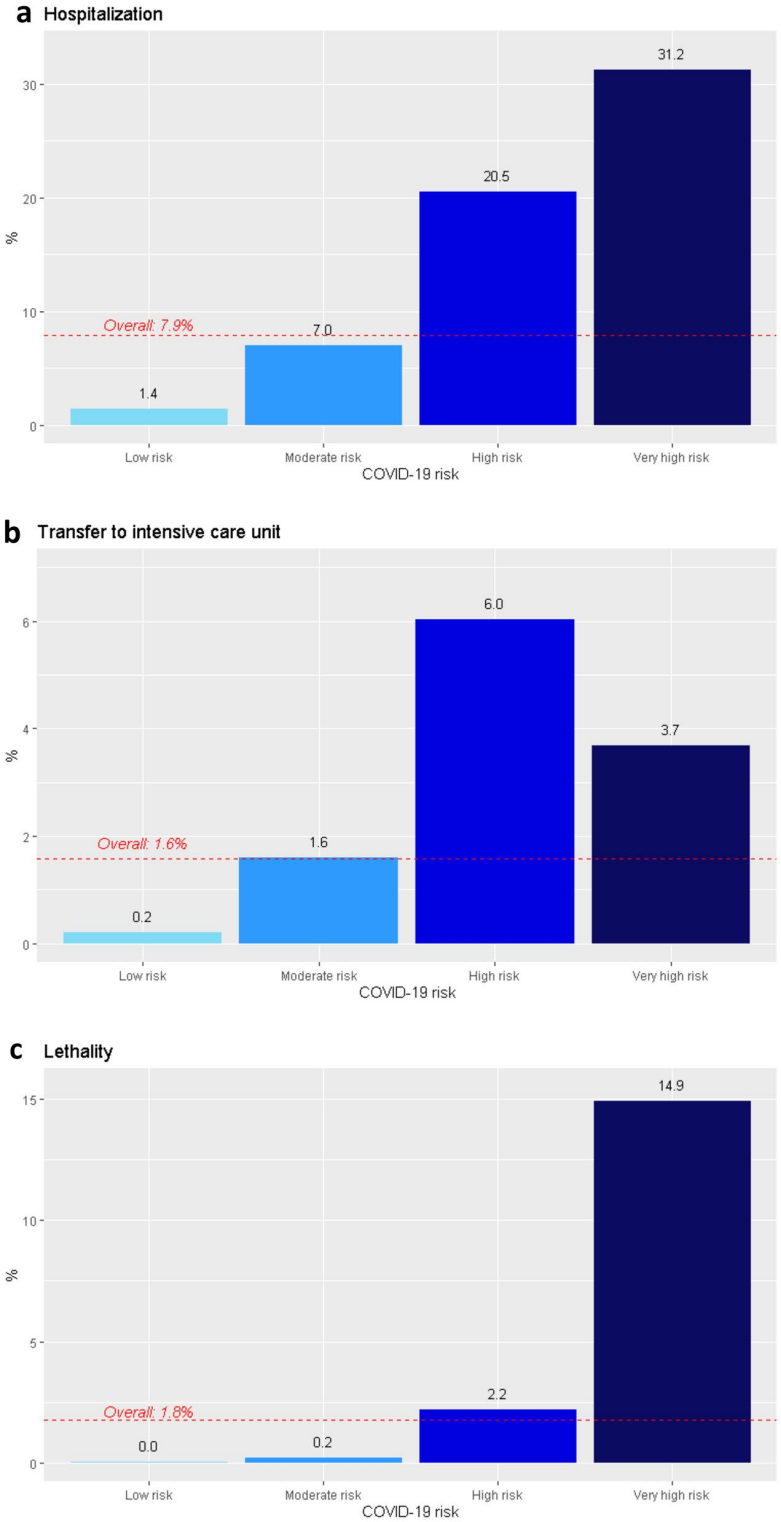
Proportion of individuals with RT-PCR-confirmed COVID-19 (*N* = 218,329) who experienced each of the events within the validation period (from September 16 to December 27, 2020). The dotted red line shows the overall event rate. (**a**) Hospital admission (*n* = 17,235). (**b**) Transfer to an intensive care unit (ICU) (*n* = 3,450). (**c**) Lethality (*n* = 3,852).

The independent dataset for model validation included 218,329 individuals with RT-PCR-confirmed COVID-19 diagnosis. Of these, 17,235 were admitted to hospital during the validation period, 3450 were transferred to the ICU, and 3,852 died. The incidence rates over COVID-19 cases (in events per 1000 persons per year) were 284.8 for hospitalization, 57.0 for ICU admission, and 63.6 for death. Figure 4 shows the incidence rate of each outcome among individuals infected during the validation period. Hospitalization rate among infected individuals progressively increased across risk groups (Fig. 4a). The rate of ICU transfer was higher in the high-risk group than the very high-risk group (Fig. 4b). Lethality progressively increased from the low-risk to the high-risk group and sharply increased in the very high-risk group (Fig. 4c). AUC ROC (95% CI) for hospitalization, ICU transfer, and death within the validation period was 0.85 (0.85–0.85), 0.86 (0.86–0.97), and 0.96 (0.96–0.96), respectively.

## Discussion

We designed a population-based risk model aimed at stratifying the general population into mutually exclusive groups at risk of COVID-19 severe illness or death. The model showed adequate goodness of fit for hospital admissions, ICU transfers, and death. When tested on an independent dataset of RT-PCR-confirmed COVID-19 individuals, the stratification model showed high discrimination capacity for the three outcomes. The highest differences between risk groups were observed for hospitalization rate. The frequency of ICU transfer was higher in the high-risk group than in the very high-risk group, probably because older and more frail people, typically in the very high-risk group, are often excluded from invasive practices to prevent therapeutic obstinacy. The mortality rate was notably higher in the very high-risk group than in other risk groups.

The living systematic review of the COVID-19 precise consortium identified 107 prognostic models for patients with COVID-19 diagnosis^11^. However, most of these models target individuals admitted to hospital or presenting at general practitioner with symptoms suspicious of COVID-19. Alternatively, we used data from the general population to develop a model that could provide a risk estimate, irrespective of the disease stage. This feature is important for prioritizing interventions and resources for people at risk of more severe outcomes in the event of SARS-CoV-2 infection. Using a population-based approach, DeCapprio et al. modelled nationwide data to develop an index to predict complications due to upper respiratory infections (as a proxy for vulnerability to COVID-19) among the general population^12^. The model showed a AUROC 0.81, close to that found in our analysis using nationwide data from COVID-19 patients (i.e., 0.85, 0.86, and 0.96 for hospital admission, ICU transfer, and death, respectively). However, the model was not COVID-19-specific and did not allow allocating individuals into risk groups. Alternatively, more in line with our approach, Mancilla-Galindo et al. developed a scoring system with high discrimination capacity for death (Harrell's C-index 0.8; 95% CI 0.796–0.804) that allowed establishing risk groups based on score cut-offs^14^. This approach of risk groups, also sought in our work, offers policymakers of countries with centralized healthcare databases a helpful tool for prioritizing resources under a “stratify-and-shield” strategy. The discrimination capacity of our model when applied to an independent dataset of RT-PCR-confirmed COVID-19 patients infected after the development period indicates that the model is also suitable for supporting therapeutic decision-making when managing COVID-19 cases.

In line with previous (i.e., early and recent) analysis of COVID-19 risk^9,10,26,27^, we found that age was the most important factor for predicting mortality, and that age and underlying conditions such as diabetes, arterial hypertension, and cardiovascular diseases significantly contributed to the risk of severe disease outcomes, particularly hospital admission. However, rather than individual diagnoses, the comorbidity burden was a strong predictor of hospital admissions and deaths. Of note, unlike variables such as age or a particular diagnosis, which are homogeneous across countries and studies, the measurement of the comorbidity burden is challenged by the lack of consensus for defining and weighting chronic conditions to be considered^28,29^. In the model by Mancilla-Galindo et al., the comorbidity burden was represented by a list of six relevant chronic conditions^14^. Alternatively, we used a multimorbidity stratification tool that had shown high accuracy in predicting the use of healthcare services, including hospital admission^30,31^. Although the use of other multimorbidity measures may change the performance of the model, the remarkable weight of this factor encourage the use of exhaustive measures of multimorbidity for health risk assessment in the COVID-19 setting.

Our analysis was strengthened by the consistent performance of the model in two different periods and independent datasets. We deemed the analysis covering the two waves important because the overburdening of the healthcare system and resource shortage experienced during the first wave (i.e., when the model was developed) might act as important confounders. Data collection during this period was also hampered by the unavailability of RT-PCR tests in some diagnoses, which were reported in electronic health records based on other criteria. Of note, this was not the case for the validation period, in which all COVID-19 cases were RT-PCR-confirmed. Another strength was the possibility of using data from the entire population. On the other hand, the use of administrative databases of data collected in routine care may limit the inclusion of all variables with potential influence on severe illness or death. Some of these variables (e.g., inflammatory biomarkers on admission, associated with poorer outcome^32^) can only be collected after COVID-19 onset and were clearly inadequate for a risk model aimed at stratifying the entire population based on baseline data stored in electronic health records. Conversely, other variables such as the blood group, with proven influence on COVID-19 outcome^33,34^, is a feature that can be known before COVID-19 onset and could be, therefore, included to the model if available; unfortunately, this variable is not routinely included in the source databases of our analysis and could not be added to the model. This limitation is common among many prediction models proposed for COVID-19^11^. Another potential limitation of our findings is the evolving context of new SARS-CoV-2 variants. Of note, data used for model development was corresponded to a mixture of variants, whereas model validation was performed during a period in which the alpha variant was dominant. These results, together with available evidence suggesting minimal differences in hospitalization rates between variants, suggest little impact of the SARS-CoV-2 variant on risk stratification. Nevertheless, we cannot rule out that future variants may limit the generalizability of the results and would require recalibration of the model. Likewise, the risk reduction for severe disease conferred by vaccines is not homogeneous between individual profiles. Therefore, although we expect age and comorbidities to play an important role, regardless of the vaccination status, future analyses shall revise and re-define risk groups in the post-vaccination context. Also, future studies on risk models should include the follow-up time perspective, not considered in our analysis.

It is worth mentioning that one of the most important components of the model is an exhaustive measure of multimorbidity developed in our area, which may limit the straight application to other countries. However, although the GMA is not yet publicly available, as a non-commercial tool, it has been freely transferred to other countries for research purposes and is available from the corresponding author upon request. The essential information for the GMA tool to be used includes individual data (i.e., identification number, birth date, and sex) and a list of all his/her diagnoses (and/or relevant health conditions), along with the type of diagnostic classification used (the system accepts ICD-9 CM, ICD-10, ICD-10-CM, CIAP-1, and CIAP-2) and the date of diagnosis^23^. Alternatively, the use of other case-mix tools with similar characteristics (e.g., Adjusted Clinical Groups^35^ and Clinical Risk Groups^36^) could be also explored. Irrespective of the multimorbidity measure used, the proposed stratification system requires a centralized collection and management of health information from the entire (or nearly) population.

In summary, the proposed risk stratification model for COVID-19 provides policymakers of countries with systematic collection of health information with evidence-based criteria for prioritizing limited COVID-19 resources, including vaccines, treatments, and tests for preventive screening of the general population. This model can also be used for needs planning (e.g., hospital and ICU beds) and, to a lesser extent, to support clinicians with dynamic risk assessment of newly diagnosed COVID-19 patients. Future analyses shall update the model to the context of vaccination and dominance of other variants of concern such as the Omicron. Of note, when prioritizing healthcare resources, other criteria aside from health risk shall be considered, including high exposure to SARS-CoV-2 and the development strategic actions for pandemic containment.

## Supplementary Information

Supplementary Information.

## Data Availability

The datasets generated and/or analysed during the current study are not publicly accessible but are available from the corresponding author upon reasonable request.
