# Supplementary material for: Development and validation of a population-based risk stratification model for severe COVID-19 in the general population
Source: Sci Rep. 2022 Feb 28;12:3277. doi: 10.1038/s41598-022-07138-y (PMC8885698; doi:10.1038/s41598-022-07138-y)
Supplement: Supplementary file 1 — Supplementary Information. [file 41598_2022_7138_MOESM1_ESM.pdf]

Development and performance of a population-based risk stratification model for COVID-19

Supplementary file 1

Contents

Supplementary Tables 2

Table S1. Delta values of the Akaike Information Criteria used for variable selection 2

Table S2. Diagnostic criteria in the development dataset 4

Table S3. Full model for hospitalization 5

Table S4. Full model for admission to intensive care unit 7

Table S5. Full model for mortality 9

Supplementary Figures 11

Figure S1. Average weekly variants of SARS-CoV-2 during the study period. **A**: Pango lineages. **B**: WHO lineages 11

Figure S2. Main effects models (Poisson regression) for explaining hospital admissions (**A**), ICU transfer (**B**), and death (**C**) due to COVID-19. Models were built using data from the entire catchment population between the development period: from March 01 to September 15, 2020. Variables are listed following the sequence in which they entered to the model. For the socioeconomic status, moderate income was used as a reference due to the low number of cases in the extreme groups. 12

Figure S3. Main demographic and clinical characteristics of individuals included in each risk group: very high risk (**A, B**), high risk (**C, D**), moderate risk (**E, F**), and low risk (**G, H**). 14

Figure S4. Calibration between expected and observed cases of the stratification model when considering data gathered during the development period (i.e., March 01 to September 15, 2020). Results are presented as No. of individuals experiencing the following outcomes due to COVID-19: hospital admissions (**A**), ICU transfer (**B**), and death (**C**)........ 15

List of ICD-9-CM codes considered for the main effects model 17

Supplementary Tables

Table S1. Delta values of the Akaike Information Criteria used for variable selection

	AIC	Delta AIC
**Model for hospital admissions due to covid-19**		
Null	144,067.40	
+ Age group	107,254.90	36,812.50
+ Health risk (GMA level)	98,181.60	9,073.30
+ Nursing home resident	92,785.92	5,395.68
+ Gender	90,978.38	1,807.54
+ Obesity	90,118.23	860.15
+ Smoker	89,604.04	514.19
+ Heart failure	89,526.48	77.56
+ Active neoplasm	89,440.95	85.53
+ Diabetes mellitus	89,388.11	52.84
+ Socioeconomic status	89,338.17	49.94
+ COPD	89,298.91	39.26
+ Chronic kidney disease	89,265.87	33.04
+ Severe intellectual disability	89,244.95	20.92
+ Hyperlipidemia	89,225.06	19.89
+ Dementia	89,213.08	11.98
+ Hypertension	89,202.73	10.35
+ Ischemic heart disease	89,202.47	0.26
**Model for transfer to intensive care unit due to covid-19**		
Null	38,426.81	
+ Age group	31,459.33	6,967.48
+ Gender	30,053.27	1,406.06
+ Health risk (GMA level)	28,671.50	1,381.77
+ Obesity	28,054.56	616.94
+ Nursing home resident	27,875.99	178.57
+ Smoker	27,710.86	165.13
+ Diabetes mellitus	27,632.23	78.63
+ Dementia	27,577.33	54.90
+ Socioeconomic status	27,538.09	39.24
+ Hyperlipidemia	27,513.31	24.78
+ Chronic kidney disease	27,503.68	9.63
+ Heart failure	27,498.06	5.62
+ Hypertension	27,495.52	2.54
+ Ischemic heart disease	27,494.12	1.40
+ AIDS-HIV	27,493.30	0.82
+ COPD	27,493.05	0.25
+ Active neoplasm	27,492.91	0.14
**Model for deaths due to covid-19**		
Null	124,318.40	

+ Age group	76,403.29	47,915.11
+ Nursing home resident	62,506.31	13,896.98
+ Health risk (GMA level)	59,670.70	2,835.61
+ Gender	58,489.27	1,181.43
+ Dementia	58,115.03	374.24
+ Heart failure	58,035.95	79.08
+ Chronic kidney disease	57,989.72	46.23
+ Active neoplasm	57,954.19	35.53
+ Psychiatric chronic disease	57,919.50	34.69
+ Diabetes mellitus	57,903.48	16.02
+ Socioeconomic status	57,886.77	16.71
+ Hyperlipidemia	57,879.41	7.36
+ Stroke	57,872.68	6.73
+ Severe intellectual disability	57,866.09	6.59
+ Smoker	57,860.47	5.62
+ Hypertension	57,859.35	1.12

Table S2. Diagnostic criteria in the development dataset

	Hospitalized		ICU Admission		Death	
	No.	%	No.	%	No.	%
Positive PCR result	34312	82.7%	7253	90.8%	8822	57.8%
Antigen-detecting rapid diagnostic test	782	1.9%	72	0.9%	141	0.9%
Serologic test	408	1.0%	67	0.8%	9	0.1%
Epidemiological criteria	5965	14.4%	595	7.4%	6291	41.2%
**TOTAL**	41468		7987		15262	

Table S3. Full model for hospitalization

	beta	RR	95% CI	
Gender				
Males	0.000	1.000		
Females	-0.076	0.927	0.841	1.021
Age	0.106	1.112	1.108	1.116
Age squared	-0.001	0.999	0.999	0.999
Health risk (GMA level)				
Basal risk	0.000	1.000		
Low risk	0.732	2.079	1.847	2.340
Moderate risk	1.200	3.319	2.833	3.889
High risk	2.640	14.010	11.113	17.662
Socioeconomic status				
High	0.236	1.266	0.747	2.146
Moderate	0.000	1.000		
Low	0.410	1.506	1.373	1.653
Very low	0.270	1.310	1.058	1.622
Institutionalized in a nursing home	2.619	13.728	11.151	16.900
Obesity	1.069	2.913	2.647	3.207
Smoker	-1.688	0.185	0.162	0.211
Heart failure	0.205	1.228	1.185	1.273
Active neoplasm	0.470	1.599	1.314	1.946
Diabetes mellitus	0.412	1.510	1.304	1.750
COPD	-0.345	0.708	0.587	0.855
Chronic kidney disease	0.344	1.410	1.160	1.715
Severe intellectual disability	0.173	1.188	0.987	1.431
Hyperlipidemia	0.221	1.247	1.110	1.402
Dementia	-0.036	0.965	0.921	1.011
Hypertension	-0.090	0.914	0.890	0.939
Ischemic heart disease	-0.476	0.621	0.490	0.788
Gender Females* Age	-0.011	0.989	0.988	0.991
Gender Females* Low health risk	0.129	1.138	1.062	1.219
Gender Females* Moderate health risk	0.279	1.322	1.225	1.427
Gender Females* High health risk	0.488	1.629	1.490	1.782
Gender Females* High Socioeconomic status	-0.285	0.752	0.555	1.020
Gender Females* Low Socioeconomic status	0.119	1.126	1.074	1.181
Gender Females* Very low Socioeconomic status	-0.022	0.979	0.881	1.087
Gender Females* Institutionalized in a nursing home	0.205	1.228	1.146	1.316
Gender Females* Obesity	0.051	1.052	1.007	1.100
Gender Females* Smoker	-0.158	0.854	0.804	0.907
Gender Females* Hyperlipidemia	-0.103	0.902	0.861	0.945
Gender Females* Ischemic heart disease	0.071	1.073	0.998	1.155
Age*Low health risk	-0.006	0.994	0.992	0.996
Age*Moderate health risk	-0.009	0.991	0.989	0.994
Age*High health risk	-0.022	0.978	0.974	0.981
Age*High Socioeconomic status	-0.007	0.993	0.985	1.001

Age*Low Socioeconomic status	-0.006	0.994	0.993	0.995
Age*Very low Socioeconomic status	-0.002	0.998	0.995	1.002
Age*Institutionalized in a nursing home	-0.014	0.986	0.984	0.989
Age*Obesity	-0.012	0.988	0.987	0.990
Age*Smoker	0.021	1.022	1.020	1.023
Age*Active neoplasm	-0.005	0.995	0.993	0.998
Age*Diabetes mellitus	-0.005	0.995	0.993	0.997
Age*COPD	0.006	1.006	1.003	1.008
Age*Chronic kidney disease	-0.003	0.997	0.995	1.000
Age*Hyperlipidemia	-0.002	0.998	0.996	1.000
Age*Ischemic heart disease	0.005	1.005	1.002	1.009
(Intercept)	-9.629	0.000	0.000	0.000

Table S4. Full model for admission to intensive care unit

	beta	RR	95% CI	
Gender				
Males	0.000	1.000		
Females	0.869	2.385	1.268	4.485
Age	0.231	1.260	1.241	1.280
Age squared	-0.002	0.998	0.998	0.999
Health risk (GMA level)				
Basal risk				
Low risk	0.524	1.690	1.223	2.334
Moderate risk	1.726	5.617	3.800	8.302
High risk	3.943	51.550	30.281	87.758
Socioeconomic status				
High	1.987	7.294	2.205	24.127
Moderate				
Low	0.966	2.627	2.043	3.379
Very low	1.232	3.429	2.003	5.870
Institutionalized in a nursing home	2.231	9.309	5.005	17.313
Obesity	1.575	4.832	3.780	6.177
Smoker	-2.824	0.059	0.043	0.082
Diabetes mellitus	0.831	2.296	1.640	3.213
Dementia	1.173	3.230	0.808	12.921
Hyperlipidemia	0.124	1.132	1.074	1.193
Chronic kidney disease	0.585	1.795	1.125	2.865
Heart failure	0.230	1.258	1.120	1.414
Active neoplasm	0.073	1.076	0.992	1.167
Gender Females* Age	-0.075	0.928	0.908	0.948
Gender Females* Age squared	0.001	1.001	1.000	1.001
Gender Females* Low health risk	0.216	1.241	1.051	1.466
Gender Females* Moderate health risk	0.351	1.420	1.191	1.693
Gender Females* High health risk	0.887	2.429	1.958	3.011
Gender Females* High Socioeconomic status	-0.391	0.676	0.309	1.481
Gender Females* Low Socioeconomic status	0.176	1.193	1.060	1.342
Gender Females* Very low Socioeconomic status	-0.152	0.859	0.683	1.080
Gender Females* Obesity	0.131	1.140	1.023	1.271
Gender Females* Dementia	-0.289	0.749	0.501	1.119
Gender Females*Chronic kidney disease	0.238	1.268	1.078	1.492
Gender Females*Heart failure	-0.132	0.876	0.719	1.068
Age*Low health risk	-0.005	0.995	0.989	1.001
Age*Moderate health risk	-0.020	0.980	0.974	0.986
Age*High health risk	-0.050	0.951	0.944	0.959
Age*High Socioeconomic status	-0.034	0.967	0.947	0.986
Age*Low Socioeconomic status	-0.015	0.985	0.981	0.989
Age*Very low Socioeconomic status	-0.013	0.987	0.978	0.995

Age*Institutionalized in a nursing home	-0.016	0.984	0.976	0.993
Age*Obesity	-0.016	0.984	0.980	0.988
Age*Smoker	0.039	1.040	1.034	1.045
Age*Diabetes mellitus	-0.009	0.991	0.987	0.996
Age*Dementia	-0.022	0.979	0.961	0.997
Age*Chronic kidney disease	-0.007	0.993	0.986	0.999
(Intercept)	-14.618	0.000	0.000	0.000

Table S5. Full model for mortality

	beta	RR	95% CI	
Gender				
Males	0	1		
Females	-1.199	0.301	0.226	0.402
Age	0.118	1.125	1.109	1.142
Age squared	0.000	1.000	1.000	1.000
Health risk (GMA level)				
Basal risk	0.000	1.000		
Low risk	0.800	2.226	1.380	3.590
Moderate risk	2.529	12.540	7.868	19.987
High risk	4.851	127.925	73.549	222.501
Socioeconomic status				
High	-1.467	0.231	0.049	1.095
Moderate	0.000	1.000		
Low	0.602	1.825	1.358	2.454
Very low	1.765	5.841	3.330	10.244
Institutionalized in a nursing home	3.516	33.661	23.065	49.124
Dementia	2.021	7.545	4.767	11.943
Heart failure	0.891	2.436	1.642	3.614
Chronic kidney disease	0.741	2.099	1.471	2.996
Active neoplasm	2.063	7.870	5.369	11.537
Psychiatric chronic disease	0.108	1.114	1.061	1.169
Diabetes mellitus	0.702	2.019	1.480	2.753
Hyperlipidemia	-0.044	0.957	0.924	0.992
Stroke	0.066	1.068	1.026	1.113
Smoker	-0.401	0.669	0.490	0.914
Hypertension	-0.094	0.910	0.870	0.952
Gender Females* Age	0.006	1.006	1.002	1.009
Gender Females* Institutionalized in a nursing home	0.242	1.274	1.182	1.374
Gender Females* Diabetes mellitus	0.145	1.156	1.075	1.243
Age*Low health risk	-0.006	0.994	0.987	1.000
Age*Moderate health risk	-0.023	0.978	0.972	0.984
Age*High health risk	-0.046	0.955	0.949	0.962
Age*High Socioeconomic status	0.019	1.019	1.000	1.039
Age*Low Socioeconomic status	-0.009	0.991	0.988	0.995
Age*Very low Socioeconomic status	-0.023	0.978	0.971	0.985
Age*Institutionalized in a nursing home	-0.018	0.982	0.978	0.986
Age*Dementia	-0.018	0.982	0.977	0.987
Age*Heart failure	-0.008	0.992	0.987	0.997
Edat:IRCSi	-0.007	0.993	0.989	0.997
Age*Chronic kidney disease	-0.024	0.977	0.972	0.981
Age*Diabetes mellitus	-0.008	0.992	0.988	0.996
Age*Smoker	0.006	1.006	1.002	1.010
(Intercept)	-14.860	0.000	0.000	0.000

Supplementary Figures

Figure S1. Average weekly variants of SARS-CoV-2 during the study period. **A**: Pango lineages. **B**: WHO lineages.

A

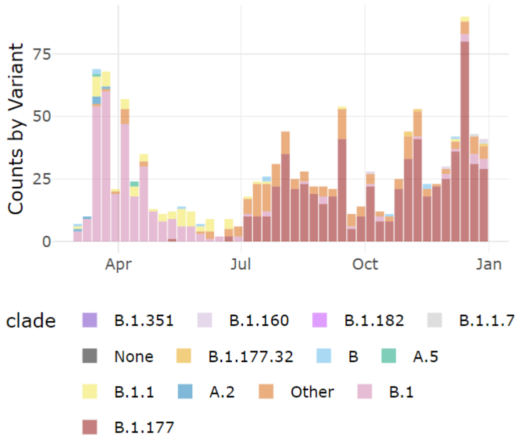

B

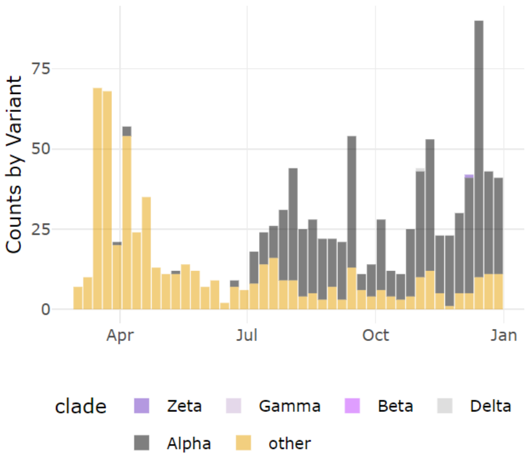

Source: http://covidtag.paseq.org/

Figure S2. Main effects models (Poisson regression) for explaining hospital admissions (**A**), ICU transfer (**B**), and death (**C**) due to COVID-19. Models were built using data from the entire catchment population between the development period: from March 01 to September 15, 2020. Variables are listed following the sequence in which they entered to the model. For the socioeconomic status, moderate income was used as a reference due to the low number of cases in the extreme groups.

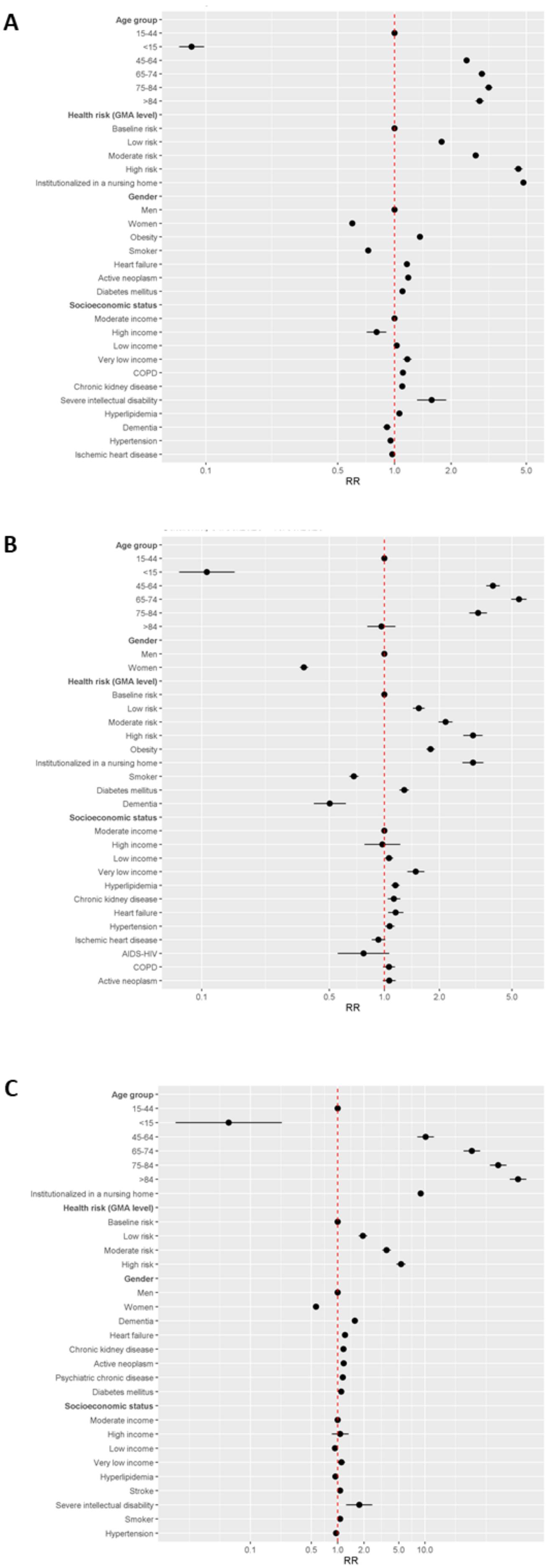
A
Age group
15-44
<15
45-64
65-74
75-84
>84
Health risk (GMA level)
Baseline risk
Low risk
Moderate risk
High risk
Institutionalized in a nursing home
Gender
Men
Women
Obesity
Smoker
Heart failure
Active neoplasm
Diabetes mellitus
Socioeconomic status
Moderate income
High income
Low income
Very low income
COPD
Chronic kidney disease
Severe intellectual disability
Hyperlipidemia
Dementia
Hypertension
Ischemic heart disease
0.1
0.5
1.0
2.0
5.0
RR
B
Age group
15-44
<15
45-64
65-74
75-84
>84
Gender
Men
Women
Health risk (GMA level)
Baseline risk
Low risk
Moderate risk
High risk
Obesity
Institutionalized in a nursing home
Smoker
Diabetes mellitus
Dementia
Socioeconomic status
Moderate income
High income
Low income
Very low income
Hyperlipidemia
Chronic kidney disease
Heart failure
Hypertension
Ischemic heart disease
AIDS-HIV
COPD
Active neoplasm
0.1
0.5
1.0
2.0
5.0
RR
C
Age group
15-44
<15
45-64
65-74
75-84
>84
Institutionalized in a nursing home
Health risk (GMA level)
Baseline risk
Low risk
Moderate risk
High risk
Gender
Men
Women
Dementia
Heart failure
Chronic kidney disease
Active neoplasm
Psychiatric chronic disease
Diabetes mellitus
Socioeconomic status
Moderate income
High income
Low income
Very low income
Hyperlipidemia
Stroke
Severe intellectual disability
Smoker
Hypertension
0.1
0.5
1.0
2.0
5.0
10.0
RR

Figure S3. Main demographic and clinical characteristics of individuals included in each risk group: very high risk (**A, B**), high risk (**C, D**), moderate risk (**E, F**), and low risk (**G, H**).

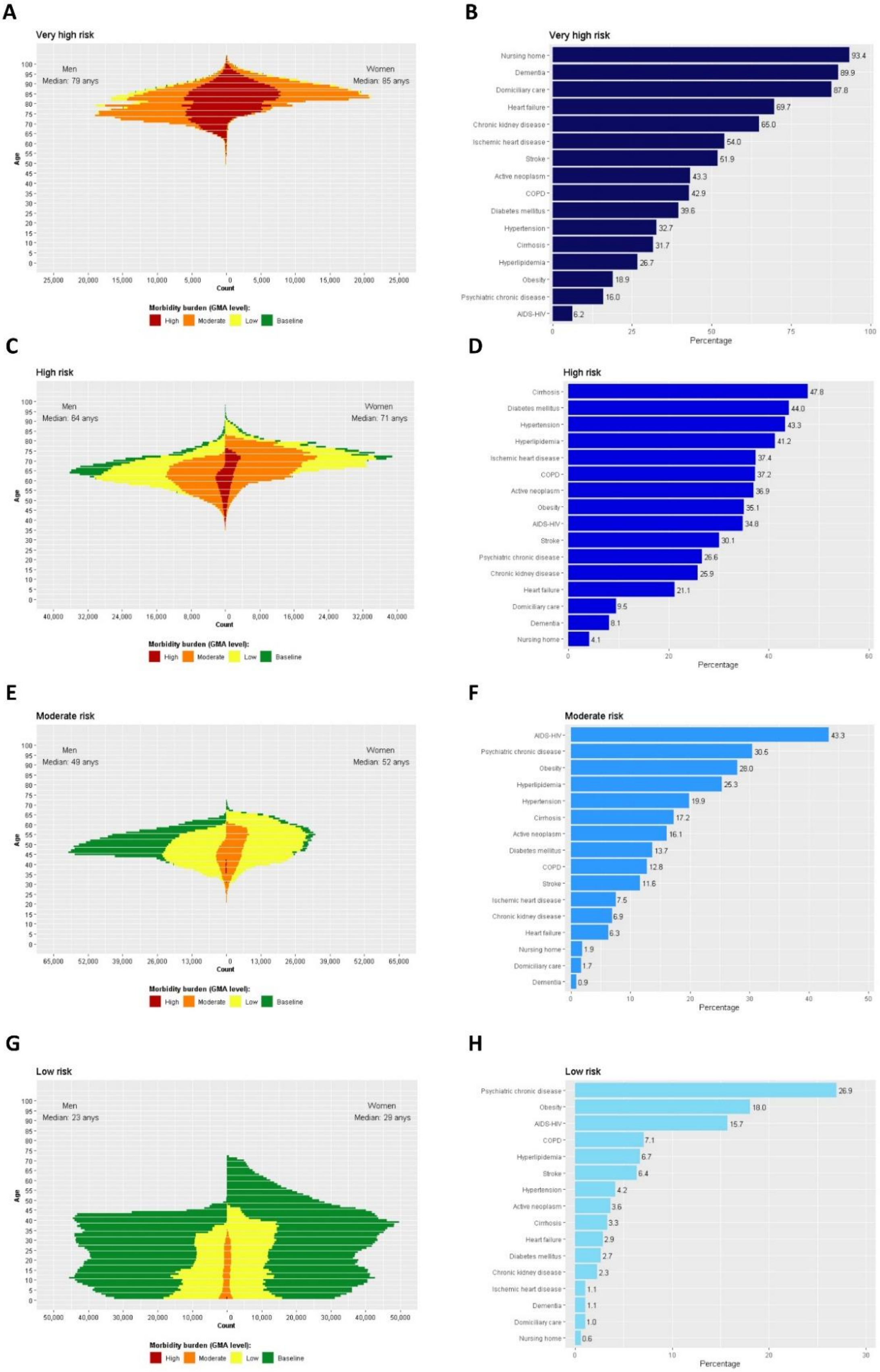

Figure S4. Calibration between expected and observed cases of the stratification model when considering data gathered during the development period (i.e., March 01 to September 15, 2020). Results are presented as No. of individuals experiencing the following outcomes due to COVID-19: hospital admissions (**A**), ICU transfer (**B**), and death (**C**).

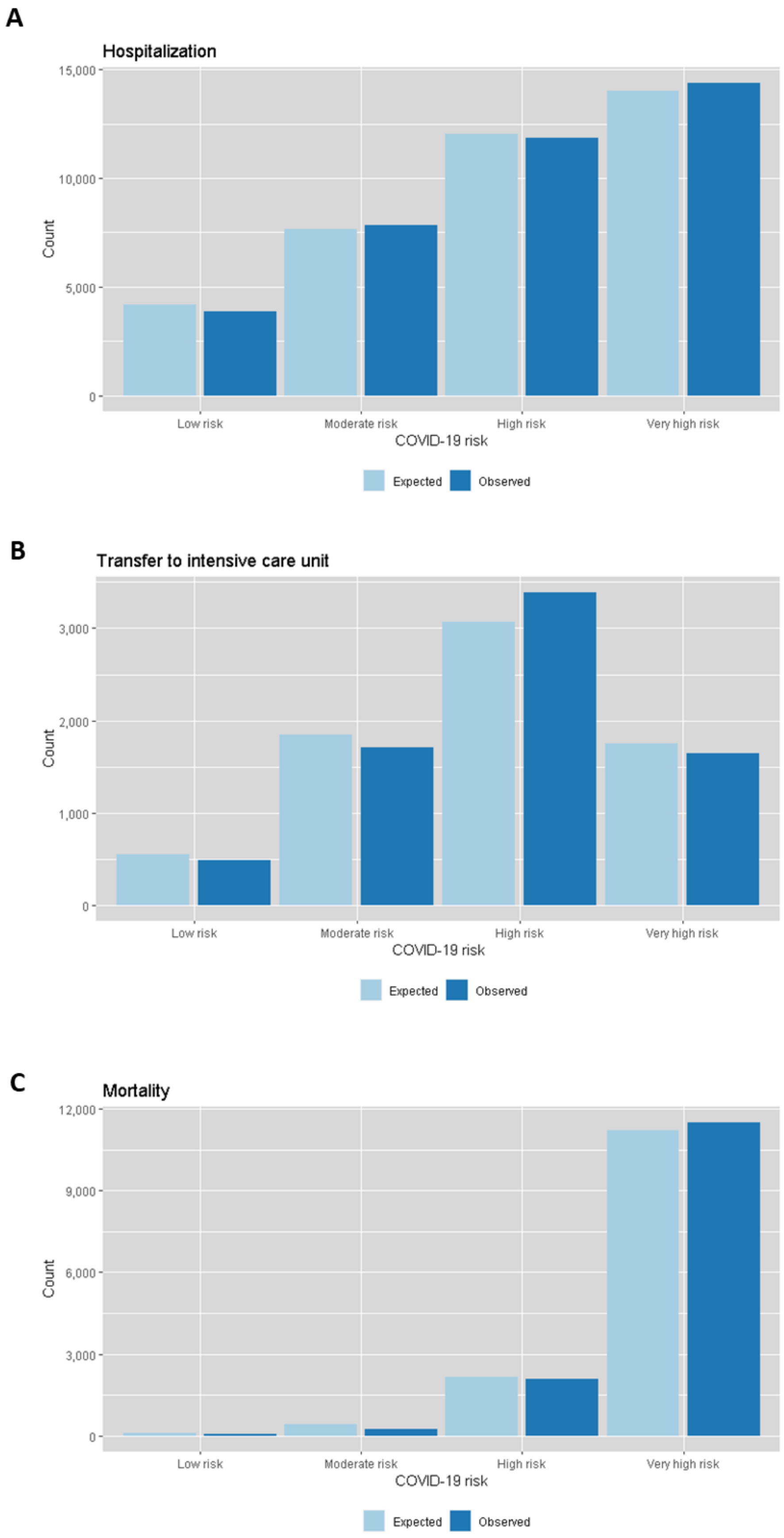
A
Hospitalization
15,000
10,000
5,000
0
Count
Low risk
Moderate risk
High risk
Very high risk
COVID-19 risk
Expected
Observed
B
Transfer to intensive care unit
3,000
2,000
1,000
0
Count
Low risk
Moderate risk
High risk
Very high risk
COVID-19 risk
Expected
Observed
C
Mortality
12,000
9,000
6,000
3,000
0
Count
Low risk
Moderate risk
High risk
Very high risk
COVID-19 risk
Expected
Observed

List of ICD-9-CM codes considered for the main effects model

Diabetes mellitus:

Code	Description
25000	Diabetes mellitus without mention of complication, type II or unspecified type, not stated as uncontrolled
25001	Diabetes mellitus without mention of complication, type I [juvenile type], not stated as uncontrolled
25002	Diabetes mellitus without mention of complication, type II or unspecified type, uncontrolled
25003	Diabetes mellitus without mention of complication, type I [juvenile type], uncontrolled
25010	Diabetes with ketoacidosis, type II or unspecified type, not stated as uncontrolled
25011	Diabetes with ketoacidosis, type I [juvenile type], not stated as uncontrolled
25012	Diabetes with ketoacidosis, type II or unspecified type, uncontrolled
25013	Diabetes with ketoacidosis, type I [juvenile type], uncontrolled
25020	Diabetes with hyperosmolarity, type II or unspecified type, not stated as uncontrolled
25021	Diabetes with hyperosmolarity, type I [juvenile type], not stated as uncontrolled
25022	Diabetes with hyperosmolarity, type II or unspecified type, uncontrolled
25023	Diabetes with hyperosmolarity, type I [juvenile type], uncontrolled
25030	Diabetes with other coma, type II or unspecified type, not stated as uncontrolled
25031	Diabetes with other coma, type I [juvenile type], not stated as uncontrolled
25032	Diabetes with other coma, type II or unspecified type, uncontrolled
25033	Diabetes with other coma, type I [juvenile type], uncontrolled
25040	Diabetes with renal manifestations, type II or unspecified type, not stated as uncontrolled
25041	Diabetes with renal manifestations, type I [juvenile type], not stated as uncontrolled
25042	Diabetes with renal manifestations, type II or unspecified type, uncontrolled
25043	Diabetes with renal manifestations, type I [juvenile type], uncontrolled
25050	Diabetes with ophthalmic manifestations, type II or unspecified type, not stated as uncontrolled
25051	Diabetes with ophthalmic manifestations, type I [juvenile type], not stated as uncontrolled
25052	Diabetes with ophthalmic manifestations, type II or unspecified type, uncontrolled
25053	Diabetes with ophthalmic manifestations, type I [juvenile type], uncontrolled
25060	Diabetes with neurological manifestations, type II or unspecified type, not stated as uncontrolled
25061	Diabetes with neurological manifestations, type I [juvenile type], not stated as uncontrolled
25062	Diabetes with neurological manifestations, type II or unspecified type, uncontrolled
25063	Diabetes with neurological manifestations, type I [juvenile type], uncontrolled
25070	Diabetes with peripheral circulatory disorders, type II or unspecified type, not stated as uncontrolled
25071	Diabetes with peripheral circulatory disorders, type I [juvenile type], not stated as uncontrolled
25072	Diabetes with peripheral circulatory disorders, type II or unspecified type, uncontrolled
25073	Diabetes with peripheral circulatory disorders, type I [juvenile type], uncontrolled
25080	Diabetes with other specified manifestations, type II or unspecified type, not stated as uncontrolled
25081	Diabetes with other specified manifestations, type I [juvenile type], not stated as uncontrolled
25082	Diabetes with other specified manifestations, type II or unspecified type, uncontrolled
25083	Diabetes with other specified manifestations, type I [juvenile type], uncontrolled
25090	Diabetes with unspecified complication, type II or unspecified type, not stated as uncontrolled
25091	Diabetes with unspecified complication, type I [juvenile type], not stated as uncontrolled
25092	Diabetes with unspecified complication, type II or unspecified type, uncontrolled
25093	Diabetes with unspecified complication, type I [juvenile type], uncontrolled

Heart failure

Code	Description
39891	Rheumatic heart failure (congestive)
40201	Malignant hypertensive heart disease with heart failure
40211	Benign hypertensive heart disease with heart failure
40291	Unspecified hypertensive heart disease with heart failure
40401	Hypertensive heart and chronic kidney disease, malignant, with heart failure and with chronic kidney disease stage I through stage IV, or unspecified
40403	Hypertensive heart and chronic kidney disease, malignant, with heart failure and with chronic kidney disease stage V or end stage renal disease
40411	Hypertensive heart and chronic kidney disease, benign, with heart failure and with chronic kidney disease stage I through stage IV, or unspecified

40413	Hypertensive heart and chronic kidney disease, benign, with heart failure and chronic kidney disease stage V or end stage renal disease
40491	Hypertensive heart and chronic kidney disease, unspecified, with heart failure and with chronic kidney disease stage I through stage IV, or unspecified
40493	Hypertensive heart and chronic kidney disease, unspecified, with heart failure and chronic kidney disease stage V or end stage renal disease
4280	Congestive heart failure, unspecified
4281	Left heart failure
42820	Systolic heart failure, unspecified
42821	Acute systolic heart failure
42822	Chronic systolic heart failure
42823	Acute on chronic systolic heart failure
42830	Diastolic heart failure, unspecified
42831	Acute diastolic heart failure
42832	Chronic diastolic heart failure
42833	Acute on chronic diastolic heart failure
42840	Combined systolic and diastolic heart failure, unspecified
42841	Acute combined systolic and diastolic heart failure
42842	Chronic combined systolic and diastolic heart failure
42843	Acute on chronic combined systolic and diastolic heart failure
4289	Heart failure, unspecified

COPD

Code	Description
4910	Simple chronic bronchitis
49120	Obstructive chronic bronchitis without exacerbation
49121	Obstructive chronic bronchitis with (acute) exacerbation
49122	Obstructive chronic bronchitis with acute bronchitis
4918	Other chronic bronchitis
4919	Unspecified chronic bronchitis
4920	Emphysematous bleb
4928	Other emphysema
4940	Bronchiectasis without acute exacerbation
4941	Bronchiectasis with acute exacerbation
496	Chronic airway obstruction, not elsewhere classified

Hypertension

Code	Description
4010	Malignant essential hypertension
4011	Benign essential hypertension
4019	Unspecified essential hypertension
40200	Malignant hypertensive heart disease without heart failure
40201	Malignant hypertensive heart disease with heart failure
40210	Benign hypertensive heart disease without heart failure
40211	Benign hypertensive heart disease with heart failure
40290	Unspecified hypertensive heart disease without heart failure
40291	Unspecified hypertensive heart disease with heart failure
40300	Hypertensive chronic kidney disease, malignant, with chronic kidney disease stage I through stage IV, or unspecified
40301	Hypertensive chronic kidney disease, malignant, with chronic kidney disease stage V or end stage renal disease
40310	Hypertensive chronic kidney disease, benign, with chronic kidney disease stage I through stage IV, or unspecified
40311	Hypertensive chronic kidney disease, benign, with chronic kidney disease stage V or end stage renal disease
40390	Hypertensive chronic kidney disease, unspecified, with chronic kidney disease stage I through stage IV, or unspecified
40391	Hypertensive chronic kidney disease, unspecified, with chronic kidney disease stage V or end stage renal disease
40400	Hypertensive heart and chronic kidney disease, malignant, without heart failure and with chronic kidney disease stage I through stage IV, or unspecified
40401	Hypertensive heart and chronic kidney disease, malignant, with heart failure and with chronic kidney disease stage I through stage IV, or unspecified
40402	Hypertensive heart and chronic kidney disease, malignant, without heart failure and with chronic kidney disease stage V or end stage renal disease
40403	Hypertensive heart and chronic kidney disease, malignant, with heart failure and with chronic kidney disease stage V or end stage renal disease

40410	Hypertensive heart and chronic kidney disease, benign, without heart failure and with chronic kidney disease stage I through stage IV, or unspecified
40411	Hypertensive heart and chronic kidney disease, benign, with heart failure and with chronic kidney disease stage I through stage IV, or unspecified
40412	Hypertensive heart and chronic kidney disease, benign, without heart failure and with chronic kidney disease stage V or end stage renal disease
40413	Hypertensive heart and chronic kidney disease, benign, with heart failure and chronic kidney disease stage V or end stage renal disease
40490	Hypertensive heart and chronic kidney disease, unspecified, without heart failure and with chronic kidney disease stage I through stage IV, or unspecified
40491	Hypertensive heart and chronic kidney disease, unspecified, with heart failure and with chronic kidney disease stage I through stage IV, or unspecified
40492	Hypertensive heart and chronic kidney disease, unspecified, without heart failure and with chronic kidney disease stage V or end stage renal disease
40493	Hypertensive heart and chronic kidney disease, unspecified, with heart failure and chronic kidney disease stage V or end stage renal disease
40501	Malignant renovascular hypertension
40509	Other malignant secondary hypertension
40511	Benign renovascular hypertension
40519	Other benign secondary hypertension
40591	Unspecified renovascular hypertension
40599	Other unspecified secondary hypertension

AIDS-HIV

Code	Description
042	Human immunodeficiency virus [HIV] disease
07953	Human immunodeficiency virus, type 2 [HIV-2]
V08	Asymptomatic human immunodeficiency virus [HIV] infection status

Ischemic heart disease

Code	Description
41000	Acute myocardial infarction of anterolateral wall, episode of care unspecified
41001	Acute myocardial infarction of anterolateral wall, initial episode of care
41002	Acute myocardial infarction of anterolateral wall, subsequent episode of care
41010	Acute myocardial infarction of other anterior wall, episode of care unspecified
41011	Acute myocardial infarction of other anterior wall, initial episode of care
41012	Acute myocardial infarction of other anterior wall, subsequent episode of care
41020	Acute myocardial infarction of inferolateral wall, episode of care unspecified
41021	Acute myocardial infarction of inferolateral wall, initial episode of care
41022	Acute myocardial infarction of inferolateral wall, subsequent episode of care
41030	Acute myocardial infarction of inferoposterior wall, episode of care unspecified
41031	Acute myocardial infarction of inferoposterior wall, initial episode of care
41032	Acute myocardial infarction of inferoposterior wall, subsequent episode of care
41040	Acute myocardial infarction of other inferior wall, episode of care unspecified
41041	Acute myocardial infarction of other inferior wall, initial episode of care
41042	Acute myocardial infarction of other inferior wall, subsequent episode of care
41050	Acute myocardial infarction of other lateral wall, episode of care unspecified
41051	Acute myocardial infarction of other lateral wall, initial episode of care
41052	Acute myocardial infarction of other lateral wall, subsequent episode of care
41060	True posterior wall infarction, episode of care unspecified
41061	True posterior wall infarction, initial episode of care
41062	True posterior wall infarction, subsequent episode of care
41070	Subendocardial infarction, episode of care unspecified
41071	Subendocardial infarction, initial episode of care
41072	Subendocardial infarction, subsequent episode of care
41080	Acute myocardial infarction of other specified sites, episode of care unspecified
41081	Acute myocardial infarction of other specified sites, initial episode of care
41082	Acute myocardial infarction of other specified sites, subsequent episode of care
41090	Acute myocardial infarction of unspecified site, episode of care unspecified
41091	Acute myocardial infarction of unspecified site, initial episode of care
41092	Acute myocardial infarction of unspecified site, subsequent episode of care
4110	Postmyocardial infarction syndrome

4111	Intermediate coronary syndrome
41181	Acute coronary occlusion without myocardial infarction
41189	Other acute and subacute forms of ischemic heart disease, other
412	Old myocardial infarction
4130	Angina decubitus
4131	Prinzmetal angina
4139	Other and unspecified angina pectoris
41400	Coronary atherosclerosis of unspecified type of vessel, native or graft
41401	Coronary atherosclerosis of native coronary artery
41402	Coronary atherosclerosis of autologous vein bypass graft
41403	Coronary atherosclerosis of nonautologous biological bypass graft
41404	Coronary atherosclerosis of artery bypass graft
41405	Coronary atherosclerosis of unspecified bypass graft
41406	Coronary atherosclerosis of native coronary artery of transplanted heart
41407	Coronary atherosclerosis of bypass graft (artery) (vein) of transplanted heart
41410	Aneurysm of heart (wall)
41411	Aneurysm of coronary vessels
41412	Dissection of coronary artery
41419	Other aneurysm of heart
4142	Chronic total occlusion of coronary artery
4143	Coronary atherosclerosis due to lipid rich plaque
4148	Other specified forms of chronic ischemic heart disease
4149	Chronic ischemic heart disease, unspecified
99603	Mechanical complication due to coronary bypass graft
V4581	Aortocoronary bypass status
V4582	Percutaneous transluminal coronary angioplasty status

Stroke

Code	Description
09487	Syphilitic ruptured cerebral aneurysm
34660	Persistent migraine aura with cerebral infarction, without mention of intractable migraine without mention of status migrainosus
34661	Persistent migraine aura with cerebral infarction, with intractable migraine, so stated, without mention of status migrainosus
34662	Persistent migraine aura with cerebral infarction, without mention of intractable migraine with status migrainosus
34663	Persistent migraine aura with cerebral infarction, with intractable migraine, so stated, with status migrainosus
430	Subarachnoid hemorrhage
431	Intracerebral hemorrhage
4320	Nontraumatic extradural hemorrhage
4321	Subdural hemorrhage
4329	Unspecified intracranial hemorrhage
43300	Occlusion and stenosis of basilar artery without mention of cerebral infarction
43301	Occlusion and stenosis of basilar artery with cerebral infarction
43310	Occlusion and stenosis of carotid artery without mention of cerebral infarction
43311	Occlusion and stenosis of carotid artery with cerebral infarction
43320	Occlusion and stenosis of vertebral artery without mention of cerebral infarction
43321	Occlusion and stenosis of vertebral artery with cerebral infarction
43330	Occlusion and stenosis of multiple and bilateral precerebral arteries without mention of cerebral infarction
43331	Occlusion and stenosis of multiple and bilateral precerebral arteries with cerebral infarction
43380	Occlusion and stenosis of other specified precerebral artery without mention of cerebral infarction
43381	Occlusion and stenosis of other specified precerebral artery with cerebral infarction
43390	Occlusion and stenosis of unspecified precerebral artery without mention of cerebral infarction
43391	Occlusion and stenosis of unspecified precerebral artery with cerebral infarction
43400	Cerebral thrombosis without mention of cerebral infarction
43401	Cerebral thrombosis with cerebral infarction
43410	Cerebral embolism without mention of cerebral infarction
43411	Cerebral embolism with cerebral infarction
43490	Cerebral artery occlusion, unspecified without mention of cerebral infarction
43491	Cerebral artery occlusion, unspecified with cerebral infarction
436	Acute, but ill-defined, cerebrovascular disease
4370	Cerebral atherosclerosis
4371	Other generalized ischemic cerebrovascular disease
4378	Other ill-defined cerebrovascular disease
4379	Unspecified cerebrovascular disease

4380	Late effects of cerebrovascular disease, cognitive deficits
43810	Late effects of cerebrovascular disease, speech and language deficit, unspecified
43811	Late effects of cerebrovascular disease, aphasia
43812	Late effects of cerebrovascular disease, dysphasia
43813	Late effects of cerebrovascular disease, dysarthria
43814	Late effects of cerebrovascular disease, fluency disorder
43819	Late effects of cerebrovascular disease, other speech and language deficits
43820	Late effects of cerebrovascular disease, hemiplegia affecting unspecified side
43821	Late effects of cerebrovascular disease, hemiplegia affecting dominant side
43822	Late effects of cerebrovascular disease, hemiplegia affecting nondominant side
43830	Late effects of cerebrovascular disease, monoplegia of upper limb affecting unspecified side
43831	Late effects of cerebrovascular disease, monoplegia of upper limb affecting dominant side
43832	Late effects of cerebrovascular disease, monoplegia of upper limb affecting nondominant side
43840	Late effects of cerebrovascular disease, monoplegia of lower limb affecting unspecified side
43841	Late effects of cerebrovascular disease, monoplegia of lower limb affecting dominant side
43842	Late effects of cerebrovascular disease, monoplegia of lower limb affecting nondominant side
43850	Late effects of cerebrovascular disease, other paralytic syndrome affecting unspecified side
43851	Late effects of cerebrovascular disease, other paralytic syndrome affecting dominant side
43852	Late effects of cerebrovascular disease, other paralytic syndrome affecting nondominant side
43853	Late effects of cerebrovascular disease, other paralytic syndrome, bilateral
4386	Late effects of cerebrovascular disease, alterations of sensations
4387	Late effects of cerebrovascular disease, disturbances of vision
43881	Other late effects of cerebrovascular disease, apraxia
43882	Other late effects of cerebrovascular disease, dysphagia
43883	Other late effects of cerebrovascular disease, facial weakness
43884	Other late effects of cerebrovascular disease, ataxia
43885	Other late effects of cerebrovascular disease, vertigo
43889	Other late effects of cerebrovascular disease
4389	Unspecified late effects of cerebrovascular disease
74781	Anomalies of cerebrovascular system
7843	Aphasia
99702	Iatrogenic cerebrovascular infarction or hemorrhage

Chronic kidney disease

Code	Description
40300	Hypertensive chronic kidney disease, malignant, with chronic kidney disease stage I through stage IV, or unspecified
40301	Hypertensive chronic kidney disease, malignant, with chronic kidney disease stage V or end stage renal disease
40310	Hypertensive chronic kidney disease, benign, with chronic kidney disease stage I through stage IV, or unspecified
40311	Hypertensive chronic kidney disease, benign, with chronic kidney disease stage V or end stage renal disease
40390	Hypertensive chronic kidney disease, unspecified, with chronic kidney disease stage I through stage IV, or unspecified
40391	Hypertensive chronic kidney disease, unspecified, with chronic kidney disease stage V or end stage renal disease
40400	Hypertensive heart and chronic kidney disease, malignant, without heart failure and with chronic kidney disease stage I through stage IV, or unspecified
40401	Hypertensive heart and chronic kidney disease, malignant, with heart failure and with chronic kidney disease stage I through stage IV, or unspecified
40402	Hypertensive heart and chronic kidney disease, malignant, without heart failure and with chronic kidney disease stage V or end stage renal disease
40403	Hypertensive heart and chronic kidney disease, malignant, with heart failure and with chronic kidney disease stage V or end stage renal disease
40410	Hypertensive heart and chronic kidney disease, benign, without heart failure and with chronic kidney disease stage I through stage IV, or unspecified
40411	Hypertensive heart and chronic kidney disease, benign, with heart failure and with chronic kidney disease stage I through stage IV, or unspecified
40412	Hypertensive heart and chronic kidney disease, benign, without heart failure and with chronic kidney disease stage V or end stage renal disease
40413	Hypertensive heart and chronic kidney disease, benign, with heart failure and chronic kidney disease stage V or end stage renal disease
40490	Hypertensive heart and chronic kidney disease, unspecified, without heart failure and with chronic kidney disease stage I through stage IV, or unspecified
40491	Hypertensive heart and chronic kidney disease, unspecified, with heart failure and with chronic kidney disease stage I through stage IV, or unspecified
40492	Hypertensive heart and chronic kidney disease, unspecified, without heart failure and with chronic kidney disease stage V or end stage renal disease
40493	Hypertensive heart and chronic kidney disease, unspecified, with heart failure and chronic kidney disease stage V or end stage renal disease
5820	Chronic glomerulonephritis with lesion of proliferative glomerulonephritis

5821	Chronic glomerulonephritis with lesion of membranous glomerulonephritis
5822	Chronic glomerulonephritis with lesion of membranoproliferative glomerulonephritis
5824	Chronic glomerulonephritis with lesion of rapidly progressive glomerulonephritis
58281	Chronic glomerulonephritis in diseases classified elsewhere
58289	Chronic glomerulonephritis with other specified pathological lesion in kidney
5829	Chronic glomerulonephritis with unspecified pathological lesion in kidney
5830	Nephritis and nephropathy, not specified as acute or chronic, with lesion of proliferative glomerulonephritis
5831	Nephritis and nephropathy, not specified as acute or chronic, with lesion of membranous glomerulonephritis
5832	Nephritis and nephropathy, not specified as acute or chronic, with lesion of membranoproliferative glomerulonephritis
5834	Nephritis and nephropathy, not specified as acute or chronic, with lesion of rapidly progressive glomerulonephritis
5836	Nephritis and nephropathy, not specified as acute or chronic, with lesion of renal cortical necrosis
5837	Nephritis and nephropathy, not specified as acute or chronic, with lesion of renal medullary necrosis
5851	Chronic kidney disease, Stage I
5852	Chronic kidney disease, Stage II (mild)
5853	Chronic kidney disease, Stage III (moderate)
5854	Chronic kidney disease, Stage IV (severe)
5855	Chronic kidney disease, Stage V
5856	End stage renal disease
5859	Chronic kidney disease, unspecified
586	Renal failure, unspecified
5880	Renal osteodystrophy
7925	Cloudy (hemodialysis) (peritoneal) dialysis effluent
V420	Kidney replaced by transplant
V4511	Renal dialysis status
V4512	Noncompliance with renal dialysis
V560	Encounter for extracorporeal dialysis
V561	Fitting and adjustment of extracorporeal dialysis catheter
V562	Fitting and adjustment of peritoneal dialysis catheter
V5631	Encounter for adequacy testing for hemodialysis
V5632	Encounter for adequacy testing for peritoneal dialysis
V568	Encounter for other dialysis

Dementia

Code	Description
2900	Senile dementia, uncomplicated
29010	Presenile dementia, uncomplicated
29011	Presenile dementia with delirium
29012	Presenile dementia with delusional features
29013	Presenile dementia with depressive features
29020	Senile dementia with delusional features
29021	Senile dementia with depressive features
2903	Senile dementia with delirium
29040	Vascular dementia, uncomplicated
29041	Vascular dementia, with delirium
29042	Vascular dementia, with delusions
29043	Vascular dementia, with depressed mood
2908	Other specified senile psychotic conditions
2909	Unspecified senile psychotic condition
29410	Dementia in conditions classified elsewhere without behavioral disturbance
29411	Dementia in conditions classified elsewhere with behavioral disturbance
3310	Alzheimer's disease
33111	Pick's disease
33119	Other frontotemporal dementia
3312	Senile degeneration of brain

Obesity

Code	Description
27800	Obesity, unspecified
27801	Morbid obesity
V8531	Body Mass Index 31.0-31.9, adult
V8532	Body Mass Index 32.0-32.9, adult

V8534	Body Mass Index 34.0-34.9, adult
V8535	Body Mass Index 35.0-35.9, adult
V8536	Body Mass Index 36.0-36.9, adult
V8537	Body Mass Index 37.0-37.9, adult
V8538	Body Mass Index 38.0-38.9, adult
V8539	Body Mass Index 39.0-39.9, adult
V8541	Body Mass Index 40.0-44.9, adult
V8542	Body Mass Index 45.0-49.9, adult
V8543	Body Mass Index 50.0-59.9, adult

Hyperlipidemia

Code	Description
2720	Pure hypercholesterolemia
2721	Pure hyperglyceridemia
2722	Mixed hyperlipidemia
2723	Hyperchylomicronemia
2724	Other and unspecified hyperlipidemia

Smoker

Code	Description
3051	Tobacco use disorder
64900	Tobacco use disorder complicating pregnancy, childbirth, or the puerperium, unspecified as to episode of care or not applicable
64901	Tobacco use disorder complicating pregnancy, childbirth, or the puerperium, delivered, with or without mention of antepartum condition
64902	Tobacco use disorder complicating pregnancy, childbirth, or the puerperium, delivered, with mention of postpartum complication
64903	Tobacco use disorder complicating pregnancy, childbirth, or the puerperium, antepartum condition or complication
64904	Tobacco use disorder complicating pregnancy, childbirth, or the puerperium, postpartum condition or complication
98984	Toxic effect of tobacco
V1582	Personal history of tobacco use

Active neoplasm

Code	Description
1400	Malignant neoplasm of upper lip, vermilion border
1401	Malignant neoplasm of lower lip, vermilion border
1403	Malignant neoplasm of upper lip, inner aspect
1404	Malignant neoplasm of lower lip, inner aspect
1405	Malignant neoplasm of lip, unspecified, inner aspect
1406	Malignant neoplasm of commissure of lip
1408	Malignant neoplasm of other sites of lip
1409	Malignant neoplasm of lip, unspecified, vermilion border
1410	Malignant neoplasm of base of tongue
1411	Malignant neoplasm of dorsal surface of tongue
1412	Malignant neoplasm of tip and lateral border of tongue
1413	Malignant neoplasm of ventral surface of tongue
1414	Malignant neoplasm of anterior two-thirds of tongue, part unspecified
1415	Malignant neoplasm of junctional zone of tongue
1416	Malignant neoplasm of lingual tonsil
1418	Malignant neoplasm of other sites of tongue
1419	Malignant neoplasm of tongue, unspecified
1420	Malignant neoplasm of parotid gland
1421	Malignant neoplasm of submandibular gland
1422	Malignant neoplasm of sublingual gland
1428	Malignant neoplasm of other major salivary glands
1429	Malignant neoplasm of salivary gland, unspecified

1430	Malignant neoplasm of upper gum
1431	Malignant neoplasm of lower gum
1438	Malignant neoplasm of other sites of gum
1439	Malignant neoplasm of gum, unspecified
1440	Malignant neoplasm of anterior portion of floor of mouth
1441	Malignant neoplasm of lateral portion of floor of mouth
1448	Malignant neoplasm of other sites of floor of mouth
1449	Malignant neoplasm of floor of mouth, part unspecified
1450	Malignant neoplasm of cheek mucosa
1451	Malignant neoplasm of vestibule of mouth
1452	Malignant neoplasm of hard palate
1453	Malignant neoplasm of soft palate
1454	Malignant neoplasm of uvula
1455	Malignant neoplasm of palate, unspecified
1456	Malignant neoplasm of retromolar area
1458	Malignant neoplasm of other specified parts of mouth
1459	Malignant neoplasm of mouth, unspecified
1460	Malignant neoplasm of tonsil
1461	Malignant neoplasm of tonsillar fossa
1462	Malignant neoplasm of tonsillar pillars (anterior) (posterior)
1463	Malignant neoplasm of vallecula epiglottica
1464	Malignant neoplasm of anterior aspect of epiglottis
1465	Malignant neoplasm of junctional region of oropharynx
1466	Malignant neoplasm of lateral wall of oropharynx
1467	Malignant neoplasm of posterior wall of oropharynx
1468	Malignant neoplasm of other specified sites of oropharynx
1469	Malignant neoplasm of oropharynx, unspecified site
1470	Malignant neoplasm of superior wall of nasopharynx
1471	Malignant neoplasm of posterior wall of nasopharynx
1472	Malignant neoplasm of lateral wall of nasopharynx
1473	Malignant neoplasm of anterior wall of nasopharynx
1478	Malignant neoplasm of other specified sites of nasopharynx
1479	Malignant neoplasm of nasopharynx, unspecified site
1480	Malignant neoplasm of postcricoid region of hypopharynx
1481	Malignant neoplasm of pyriform sinus
1482	Malignant neoplasm of aryepiglottic fold, hypopharyngeal aspect
1483	Malignant neoplasm of posterior hypopharyngeal wall
1488	Malignant neoplasm of other specified sites of hypopharynx
1489	Malignant neoplasm of hypopharynx, unspecified site
1490	Malignant neoplasm of pharynx, unspecified
1491	Malignant neoplasm of waldeyer's ring
1498	Malignant neoplasm of other sites within the lip and oral cavity
1499	Malignant neoplasm of ill-defined sites within the lip and oral cavity
1500	Malignant neoplasm of cervical esophagus
1501	Malignant neoplasm of thoracic esophagus
1502	Malignant neoplasm of abdominal esophagus
1503	Malignant neoplasm of upper third of esophagus
1504	Malignant neoplasm of middle third of esophagus
1505	Malignant neoplasm of lower third of esophagus
1508	Malignant neoplasm of other specified part of esophagus
1509	Malignant neoplasm of esophagus, unspecified site
1510	Malignant neoplasm of cardia
1511	Malignant neoplasm of pylorus
1512	Malignant neoplasm of pyloric antrum
1513	Malignant neoplasm of fundus of stomach
1514	Malignant neoplasm of body of stomach
1515	Malignant neoplasm of lesser curvature of stomach, unspecified
1516	Malignant neoplasm of greater curvature of stomach, unspecified
1518	Malignant neoplasm of other specified sites of stomach
1519	Malignant neoplasm of stomach, unspecified site
1520	Malignant neoplasm of duodenum
1521	Malignant neoplasm of jejunum
1522	Malignant neoplasm of ileum
1523	Malignant neoplasm of Meckel's diverticulum
1528	Malignant neoplasm of other specified sites of small intestine
1529	Malignant neoplasm of small intestine, unspecified site
1530	Malignant neoplasm of hepatic flexure

1531	Malignant neoplasm of transverse colon
1532	Malignant neoplasm of descending colon
1533	Malignant neoplasm of sigmoid colon
1534	Malignant neoplasm of cecum
1535	Malignant neoplasm of appendix vermiformis
1536	Malignant neoplasm of ascending colon
1537	Malignant neoplasm of splenic flexure
1538	Malignant neoplasm of other specified sites of large intestine
1539	Malignant neoplasm of colon, unspecified site
1540	Malignant neoplasm of rectosigmoid junction
1541	Malignant neoplasm of rectum
1542	Malignant neoplasm of anal canal
1543	Malignant neoplasm of anus, unspecified site
1548	Malignant neoplasm of other sites of rectum, rectosigmoid junction, and anus
1550	Malignant neoplasm of liver, primary
1551	Malignant neoplasm of intrahepatic bile ducts
1552	Malignant neoplasm of liver, not specified as primary or secondary
1560	Malignant neoplasm of gallbladder
1561	Malignant neoplasm of extrahepatic bile ducts
1562	Malignant neoplasm of ampulla of vater
1568	Malignant neoplasm of other specified sites of gallbladder and extrahepatic bile ducts
1569	Malignant neoplasm of biliary tract, part unspecified site
1570	Malignant neoplasm of head of pancreas
1571	Malignant neoplasm of body of pancreas
1572	Malignant neoplasm of tail of pancreas
1573	Malignant neoplasm of pancreatic duct
1574	Malignant neoplasm of islets of langerhans
1578	Malignant neoplasm of other specified sites of pancreas
1579	Malignant neoplasm of pancreas, part unspecified
1580	Malignant neoplasm of retroperitoneum
1588	Malignant neoplasm of specified parts of peritoneum
1589	Malignant neoplasm of peritoneum, unspecified
1590	Malignant neoplasm of intestinal tract, part unspecified
1591	Malignant neoplasm of spleen, not elsewhere classified
1598	Malignant neoplasm of other sites of digestive system and intra-abdominal organs
1599	Malignant neoplasm of ill-defined sites within the digestive organs and peritoneum
1600	Malignant neoplasm of nasal cavities
1601	Malignant neoplasm of auditory tube, middle ear, and mastoid air cells
1602	Malignant neoplasm of maxillary sinus
1603	Malignant neoplasm of ethmoidal sinus
1604	Malignant neoplasm of frontal sinus
1605	Malignant neoplasm of sphenoidal sinus
1608	Malignant neoplasm of other accessory sinuses
1609	Malignant neoplasm of accessory sinus, unspecified
1610	Malignant neoplasm of glottis
1611	Malignant neoplasm of supraglottis
1612	Malignant neoplasm of subglottis
1613	Malignant neoplasm of laryngeal cartilages
1618	Malignant neoplasm of other specified sites of larynx
1619	Malignant neoplasm of larynx, unspecified
1620	Malignant neoplasm of trachea
1622	Malignant neoplasm of main bronchus
1623	Malignant neoplasm of upper lobe, bronchus or lung
1624	Malignant neoplasm of middle lobe, bronchus or lung
1625	Malignant neoplasm of lower lobe, bronchus or lung
1628	Malignant neoplasm of other parts of bronchus or lung
1629	Malignant neoplasm of bronchus and lung, unspecified
1630	Malignant neoplasm of parietal pleura
1631	Malignant neoplasm of visceral pleura
1638	Malignant neoplasm of other specified sites of pleura
1639	Malignant neoplasm of pleura, unspecified
1640	Malignant neoplasm of thymus
1641	Malignant neoplasm of heart
1642	Malignant neoplasm of anterior mediastinum
1643	Malignant neoplasm of posterior mediastinum
1648	Malignant neoplasm of other parts of mediastinum
1649	Malignant neoplasm of mediastinum, part unspecified

1650	Malignant neoplasm of upper respiratory tract, part unspecified
1658	Malignant neoplasm of other sites within the respiratory system and intrathoracic organs
1659	Malignant neoplasm of ill-defined sites within the respiratory system
1700	Malignant neoplasm of bones of skull and face, except mandible
1701	Malignant neoplasm of mandible
1702	Malignant neoplasm of vertebral column, excluding sacrum and coccyx
1703	Malignant neoplasm of ribs, sternum, and clavicle
1704	Malignant neoplasm of scapula and long bones of upper limb
1705	Malignant neoplasm of short bones of upper limb
1706	Malignant neoplasm of pelvic bones, sacrum, and coccyx
1707	Malignant neoplasm of long bones of lower limb
1708	Malignant neoplasm of short bones of lower limb
1709	Malignant neoplasm of bone and articular cartilage, site unspecified
1710	Malignant neoplasm of connective and other soft tissue of head, face, and neck
1712	Malignant neoplasm of connective and other soft tissue of upper limb, including shoulder
1713	Malignant neoplasm of connective and other soft tissue of lower limb, including hip
1714	Malignant neoplasm of connective and other soft tissue of thorax
1715	Malignant neoplasm of connective and other soft tissue of abdomen
1716	Malignant neoplasm of connective and other soft tissue of pelvis
1717	Malignant neoplasm of connective and other soft tissue of trunk, unspecified
1718	Malignant neoplasm of other specified sites of connective and other soft tissue
1719	Malignant neoplasm of connective and other soft tissue, site unspecified
1720	Malignant melanoma of skin of lip
1721	Malignant melanoma of skin of eyelid, including canthus
1722	Malignant melanoma of skin of ear and external auditory canal
1723	Malignant melanoma of skin of other and unspecified parts of face
1724	Malignant melanoma of skin of scalp and neck
1725	Malignant melanoma of skin of trunk, except scrotum
1726	Malignant melanoma of skin of upper limb, including shoulder
1727	Malignant melanoma of skin of lower limb, including hip
1728	Malignant melanoma of other specified sites of skin
1729	Melanoma of skin, site unspecified
1740	Malignant neoplasm of nipple and areola of female breast
1741	Malignant neoplasm of central portion of female breast
1742	Malignant neoplasm of upper-inner quadrant of female breast
1743	Malignant neoplasm of lower-inner quadrant of female breast
1744	Malignant neoplasm of upper-outer quadrant of female breast
1745	Malignant neoplasm of lower-outer quadrant of female breast
1746	Malignant neoplasm of axillary tail of female breast
1748	Malignant neoplasm of other specified sites of female breast
1749	Malignant neoplasm of breast (female), unspecified
1750	Malignant neoplasm of nipple and areola of male breast
1759	Malignant neoplasm of other and unspecified sites of male breast
1760	Kaposi's sarcoma, skin
1761	Kaposi's sarcoma, soft tissue
1762	Kaposi's sarcoma, palate
1763	Kaposi's sarcoma, gastrointestinal sites
1764	Kaposi's sarcoma, lung
1765	Kaposi's sarcoma, lymph nodes
1768	Kaposi's sarcoma, other specified sites
1769	Kaposi's sarcoma, unspecified site
179	Malignant neoplasm of uterus, part unspecified
1800	Malignant neoplasm of endocervix
1801	Malignant neoplasm of exocervix
1808	Malignant neoplasm of other specified sites of cervix
1809	Malignant neoplasm of cervix uteri, unspecified site
181	Malignant neoplasm of placenta
1820	Malignant neoplasm of corpus uteri, except isthmus
1821	Malignant neoplasm of isthmus
1828	Malignant neoplasm of other specified sites of body of uterus
1830	Malignant neoplasm of ovary
1832	Malignant neoplasm of fallopian tube
1833	Malignant neoplasm of broad ligament of uterus
1834	Malignant neoplasm of parametrium
1835	Malignant neoplasm of round ligament of uterus
1838	Malignant neoplasm of other specified sites of uterine adnexa
1839	Malignant neoplasm of uterine adnexa, unspecified site

1840	Malignant neoplasm of vagina
1841	Malignant neoplasm of labia majora
1842	Malignant neoplasm of labia minora
1843	Malignant neoplasm of clitoris
1844	Malignant neoplasm of vulva, unspecified site
1848	Malignant neoplasm of other specified sites of female genital organs
1849	Malignant neoplasm of female genital organ, site unspecified
185	Malignant neoplasm of prostate
1860	Malignant neoplasm of undescended testis
1869	Malignant neoplasm of other and unspecified testis
1871	Malignant neoplasm of prepuce
1872	Malignant neoplasm of glans penis
1873	Malignant neoplasm of body of penis
1874	Malignant neoplasm of penis, part unspecified
1875	Malignant neoplasm of epididymis
1876	Malignant neoplasm of spermatic cord
1877	Malignant neoplasm of scrotum
1878	Malignant neoplasm of other specified sites of male genital organs
1879	Malignant neoplasm of male genital organ, site unspecified
1880	Malignant neoplasm of trigone of urinary bladder
1881	Malignant neoplasm of dome of urinary bladder
1882	Malignant neoplasm of lateral wall of urinary bladder
1883	Malignant neoplasm of anterior wall of urinary bladder
1884	Malignant neoplasm of posterior wall of urinary bladder
1885	Malignant neoplasm of bladder neck
1886	Malignant neoplasm of ureteric orifice
1887	Malignant neoplasm of urachus
1888	Malignant neoplasm of other specified sites of bladder
1889	Malignant neoplasm of bladder, part unspecified
1890	Malignant neoplasm of kidney, except pelvis
1891	Malignant neoplasm of renal pelvis
1892	Malignant neoplasm of ureter
1893	Malignant neoplasm of urethra
1894	Malignant neoplasm of paraurethral glands
1898	Malignant neoplasm of other specified sites of urinary organs
1899	Malignant neoplasm of urinary organ, site unspecified
1900	Malignant neoplasm of eyeball, except conjunctiva, cornea, retina, and choroid
1901	Malignant neoplasm of orbit
1902	Malignant neoplasm of lacrimal gland
1903	Malignant neoplasm of conjunctiva
1904	Malignant neoplasm of cornea
1905	Malignant neoplasm of retina
1906	Malignant neoplasm of choroid
1907	Malignant neoplasm of lacrimal duct
1908	Malignant neoplasm of other specified sites of eye
1909	Malignant neoplasm of eye, part unspecified
1910	Malignant neoplasm of cerebrum, except lobes and ventricles
1911	Malignant neoplasm of frontal lobe
1912	Malignant neoplasm of temporal lobe
1913	Malignant neoplasm of parietal lobe
1914	Malignant neoplasm of occipital lobe
1915	Malignant neoplasm of ventricles
1916	Malignant neoplasm of cerebellum nos
1917	Malignant neoplasm of brain stem
1918	Malignant neoplasm of other parts of brain
1919	Malignant neoplasm of brain, unspecified
1920	Malignant neoplasm of cranial nerves
1921	Malignant neoplasm of cerebral meninges
1922	Malignant neoplasm of spinal cord
1923	Malignant neoplasm of spinal meninges
1928	Malignant neoplasm of other specified sites of nervous system
1929	Malignant neoplasm of nervous system, part unspecified
193	Malignant neoplasm of thyroid gland
1940	Malignant neoplasm of adrenal gland
1941	Malignant neoplasm of parathyroid gland
1943	Malignant neoplasm of pituitary gland and craniopharyngeal duct
1944	Malignant neoplasm of pineal gland

1945	Malignant neoplasm of carotid body
1946	Malignant neoplasm of aortic body and other paraganglia
1948	Malignant neoplasm of other endocrine glands and related structures
1949	Malignant neoplasm of endocrine gland, site unspecified
1950	Malignant neoplasm of head, face, and neck
1951	Malignant neoplasm of thorax
1952	Malignant neoplasm of abdomen
1953	Malignant neoplasm of pelvis
1954	Malignant neoplasm of upper limb
1955	Malignant neoplasm of lower limb
1958	Malignant neoplasm of other specified sites
1960	Secondary and unspecified malignant neoplasm of lymph nodes of head, face, and neck
1961	Secondary and unspecified malignant neoplasm of intrathoracic lymph nodes
1962	Secondary and unspecified malignant neoplasm of intra-abdominal lymph nodes
1963	Secondary and unspecified malignant neoplasm of lymph nodes of axilla and upper limb
1965	Secondary and unspecified malignant neoplasm of lymph nodes of inguinal region and lower limb
1966	Secondary and unspecified malignant neoplasm of intrapelvic lymph nodes
1968	Secondary and unspecified malignant neoplasm of lymph nodes of multiple sites
1969	Secondary and unspecified malignant neoplasm of lymph nodes, site unspecified
1970	Secondary malignant neoplasm of lung
1971	Secondary malignant neoplasm of mediastinum
1972	Secondary malignant neoplasm of pleura
1973	Secondary malignant neoplasm of other respiratory organs
1974	Secondary malignant neoplasm of small intestine including duodenum
1975	Secondary malignant neoplasm of large intestine and rectum
1976	Secondary malignant neoplasm of retroperitoneum and peritoneum
1977	Malignant neoplasm of liver, secondary
1978	Secondary malignant neoplasm of other digestive organs and spleen
1980	Secondary malignant neoplasm of kidney
1981	Secondary malignant neoplasm of other urinary organs
1982	Secondary malignant neoplasm of skin
1983	Secondary malignant neoplasm of brain and spinal cord
1984	Secondary malignant neoplasm of other parts of nervous system
1985	Secondary malignant neoplasm of bone and bone marrow
1986	Secondary malignant neoplasm of ovary
1987	Secondary malignant neoplasm of adrenal gland
19881	Secondary malignant neoplasm of breast
19882	Secondary malignant neoplasm of genital organs
19889	Secondary malignant neoplasm of other specified sites
1990	Disseminated malignant neoplasm without specification of site
1991	Other malignant neoplasm without specification of site
1992	Malignant neoplasm associated with transplant organ
20000	Reticulosarcoma, unspecified site, extranodal and solid organ sites
20001	Reticulosarcoma, lymph nodes of head, face, and neck
20002	Reticulosarcoma, intrathoracic lymph nodes
20003	Reticulosarcoma, intra-abdominal lymph nodes
20004	Reticulosarcoma, lymph nodes of axilla and upper limb
20005	Reticulosarcoma, lymph nodes of inguinal region and lower limb
20006	Reticulosarcoma, intrapelvic lymph nodes
20007	Reticulosarcoma, spleen
20008	Reticulosarcoma, lymph nodes of multiple sites
20010	Lymphosarcoma, unspecified site, extranodal and solid organ sites
20011	Lymphosarcoma, lymph nodes of head, face, and neck
20012	Lymphosarcoma, intrathoracic lymph nodes
20013	Lymphosarcoma, intra-abdominal lymph nodes
20014	Lymphosarcoma, lymph nodes of axilla and upper limb
20015	Lymphosarcoma, lymph nodes of inguinal region and lower limb
20016	Lymphosarcoma, intrapelvic lymph nodes
20017	Lymphosarcoma, spleen
20018	Lymphosarcoma, lymph nodes of multiple sites
20020	Burkitt's tumor or lymphoma, unspecified site, extranodal and solid organ sites
20021	Burkitt's tumor or lymphoma, lymph nodes of head, face, and neck
20022	Burkitt's tumor or lymphoma, intrathoracic lymph nodes
20023	Burkitt's tumor or lymphoma, intra-abdominal lymph nodes
20024	Burkitt's tumor or lymphoma, lymph nodes of axilla and upper limb
20025	Burkitt's tumor or lymphoma, lymph nodes of inguinal region and lower limb
20026	Burkitt's tumor or lymphoma, intrapelvic lymph nodes

20027	Burkitt's tumor or lymphoma, spleen
20028	Burkitt's tumor or lymphoma, lymph nodes of multiple sites
20030	Marginal zone lymphoma, unspecified site, extranodal and solid organ sites
20031	Marginal zone lymphoma, lymph nodes of head, face, and neck
20032	Marginal zone lymphoma, intrathoracic lymph nodes
20033	Marginal zone lymphoma, intraabdominal lymph nodes
20034	Marginal zone lymphoma, lymph nodes of axilla and upper limb
20035	Marginal zone lymphoma, lymph nodes of inguinal region and lower limb
20036	Marginal zone lymphoma, intrapelvic lymph nodes
20037	Marginal zone lymphoma, spleen
20038	Marginal zone lymphoma, lymph nodes of multiple sites
20040	Mantle cell lymphoma, unspecified site, extranodal and solid organ sites
20041	Mantle cell lymphoma, lymph nodes of head, face, and neck
20042	Mantle cell lymphoma, intrathoracic lymph nodes
20043	Mantle cell lymphoma, intra-abdominal lymph nodes
20044	Mantle cell lymphoma, lymph nodes of axilla and upper limb
20045	Mantle cell lymphoma, lymph nodes of inguinal region and lower limb
20046	Mantle cell lymphoma, intrapelvic lymph nodes
20047	Mantle cell lymphoma, spleen
20048	Mantle cell lymphoma, lymph nodes of multiple sites
20050	Primary central nervous system lymphoma, unspecified site, extranodal and solid organ sites
20051	Primary central nervous system lymphoma, lymph nodes of head, face, and neck
20052	Primary central nervous system lymphoma, intrathoracic lymph nodes
20053	Primary central nervous system lymphoma, intra-abdominal lymph nodes
20054	Primary central nervous system lymphoma, lymph nodes of axilla and upper limb
20055	Primary central nervous system lymphoma, lymph nodes of inguinal region and lower limb
20056	Primary central nervous system lymphoma, intrapelvic lymph nodes
20057	Primary central nervous system lymphoma, spleen
20058	Primary central nervous system lymphoma, lymph nodes of multiple sites
20060	Anaplastic large cell lymphoma, unspecified site, extranodal and solid organ sites
20061	Anaplastic large cell lymphoma, lymph nodes of head, face, and neck
20062	Anaplastic large cell lymphoma, intrathoracic lymph nodes
20063	Anaplastic large cell lymphoma, intra-abdominal lymph nodes
20064	Anaplastic large cell lymphoma, lymph nodes of axilla and upper limb
20065	Anaplastic large cell lymphoma, lymph nodes of inguinal region and lower limb
20066	Anaplastic large cell lymphoma, intrapelvic lymph nodes
20067	Anaplastic large cell lymphoma, spleen
20068	Anaplastic large cell lymphoma, lymph nodes of multiple sites
20070	Large cell lymphoma, unspecified site, extranodal and solid organ sites
20071	Large cell lymphoma, lymph nodes of head, face, and neck
20072	Large cell lymphoma, intrathoracic lymph nodes
20073	Large cell lymphoma, intra-abdominal lymph nodes
20074	Large cell lymphoma, lymph nodes of axilla and upper limb
20075	Large cell lymphoma, lymph nodes of inguinal region and lower limb
20076	Large cell lymphoma, intrapelvic lymph nodes
20077	Large cell lymphoma, spleen
20078	Large cell lymphoma, lymph nodes of multiple sites
20080	Other named variants of lymphosarcoma and reticulosarcoma, unspecified site, extranodal and solid organ sites
20081	Other named variants of lymphosarcoma and reticulosarcoma, lymph nodes of head, face, and neck
20082	Other named variants of lymphosarcoma and reticulosarcoma,intrathoracic lymph nodes
20083	Other named variants of lymphosarcoma and reticulosarcoma, intra-abdominal lymph nodes
20084	Other named variants of lymphosarcoma and reticulosarcoma, lymph nodes of axilla and upper limb
20085	Other named variants of lymphosarcoma and reticulosarcoma, lymph nodes of inguinal region and lower limb
20086	Other named variants of lymphosarcoma and reticulosarcoma, intrapelvic lymph nodes
20087	Other named variants of lymphosarcoma and reticulosarcoma, spleen
20088	Other named variants of lymphosarcoma and reticulosarcoma, lymph nodes of multiple sites
20100	Hodgkin's paragranuloma, unspecified site, extranodal and solid organ sites
20101	Hodgkin's paragranuloma, lymph nodes of head, face, and neck
20102	Hodgkin's paragranuloma, intrathoracic lymph nodes
20103	Hodgkin's paragranuloma, intra-abdominal lymph nodes
20104	Hodgkin's paragranuloma, lymph nodes of axilla and upper limb
20105	Hodgkin's paragranuloma, lymph nodes of inguinal region and lower limb
20106	Hodgkin's paragranuloma, intrapelvic lymph nodes
20107	Hodgkin's paragranuloma, spleen
20108	Hodgkin's paragranuloma, lymph nodes of multiple sites
20110	Hodgkin's granuloma, unspecified site, extranodal and solid organ sites
20111	Hodgkin's granuloma, lymph nodes of head, face, and neck

20112	Hodgkin's granuloma, intrathoracic lymph nodes
20113	Hodgkin's granuloma, intra-abdominal lymph nodes
20114	Hodgkin's granuloma, lymph nodes of axilla and upper limb
20115	Hodgkin's granuloma, lymph nodes of inguinal region and lower limb
20116	Hodgkin's granuloma, intrapelvic lymph nodes
20117	Hodgkin's granuloma, spleen
20118	Hodgkin's granuloma, lymph nodes of multiple sites
20120	Hodgkin's sarcoma, unspecified site, extranodal and solid organ sites
20121	Hodgkin's sarcoma, lymph nodes of head, face, and neck
20122	Hodgkin's sarcoma, intrathoracic lymph nodes
20123	Hodgkin's sarcoma, intra-abdominal lymph nodes
20124	Hodgkin's sarcoma, lymph nodes of axilla and upper limb
20125	Hodgkin's sarcoma, lymph nodes of inguinal region and lower limb
20126	Hodgkin's sarcoma, intrapelvic lymph nodes
20127	Hodgkin's sarcoma, spleen
20128	Hodgkin's sarcoma, lymph nodes of multiple sites
20140	Hodgkin's disease, lymphocytic-histiocytic predominance, unspecified site, extranodal and solid organ sites
20141	Hodgkin's disease, lymphocytic-histiocytic predominance, lymph nodes of head, face, and neck
20142	Hodgkin's disease, lymphocytic-histiocytic predominance, intrathoracic lymph nodes
20143	Hodgkin's disease, lymphocytic-histiocytic predominance, intra-abdominal lymph nodes
20144	Hodgkin's disease, lymphocytic-histiocytic predominance, lymph nodes of axilla and upper limb
20145	Hodgkin's disease, lymphocytic-histiocytic predominance, lymph nodes of inguinal region and lower limb
20146	Hodgkin's disease, lymphocytic-histiocytic predominance, intrapelvic lymph nodes
20147	Hodgkin's disease, lymphocytic-histiocytic predominance, spleen
20148	Hodgkin's disease, lymphocytic-histiocytic predominance, lymph nodes of multiple sites
20150	Hodgkin's disease, nodular sclerosis, unspecified site, extranodal and solid organ sites
20151	Hodgkin's disease, nodular sclerosis, lymph nodes of head, face, and neck
20152	Hodgkin's disease, nodular sclerosis, intrathoracic lymph nodes
20153	Hodgkin's disease, nodular sclerosis, intra-abdominal lymph nodes
20154	Hodgkin's disease, nodular sclerosis, lymph nodes of axilla and upper limb
20155	Hodgkin's disease, nodular sclerosis, lymph nodes of inguinal region and lower limb
20156	Hodgkin's disease, nodular sclerosis, intrapelvic lymph nodes
20157	Hodgkin's disease, nodular sclerosis, spleen
20158	Hodgkin's disease, nodular sclerosis, lymph nodes of multiple sites
20160	Hodgkin's disease, mixed cellularity, unspecified site, extranodal and solid organ sites
20161	Hodgkin's disease, mixed cellularity, lymph nodes of head, face, and neck
20162	Hodgkin's disease, mixed cellularity, intrathoracic lymph nodes
20163	Hodgkin's disease, mixed cellularity, intra-abdominal lymph nodes
20164	Hodgkin's disease, mixed cellularity, lymph nodes of axilla and upper limb
20165	Hodgkin's disease, mixed cellularity, lymph nodes of inguinal region and lower limb
20166	Hodgkin's disease, mixed cellularity, intrapelvic lymph nodes
20167	Hodgkin's disease, mixed cellularity, spleen
20168	Hodgkin's disease, mixed cellularity, lymph nodes of multiple sites
20170	Hodgkin's disease, lymphocytic depletion, unspecified site, extranodal and solid organ sites
20171	Hodgkin's disease, lymphocytic depletion, lymph nodes of head, face, and neck
20172	Hodgkin's disease, lymphocytic depletion, intrathoracic lymph nodes
20173	Hodgkin's disease, lymphocytic depletion, intra-abdominal lymph nodes
20174	Hodgkin's disease, lymphocytic depletion, lymph nodes of axilla and upper limb
20175	Hodgkin's disease, lymphocytic depletion, lymph nodes of inguinal region and lower limb
20176	Hodgkin's disease, lymphocytic depletion, intrapelvic lymph nodes
20177	Hodgkin's disease, lymphocytic depletion, spleen
20178	Hodgkin's disease, lymphocytic depletion, lymph nodes of multiple sites
20190	Hodgkin's disease, unspecified type, unspecified site, extranodal and solid organ sites
20191	Hodgkin's disease, unspecified type, lymph nodes of head, face, and neck
20192	Hodgkin's disease, unspecified type, intrathoracic lymph nodes
20193	Hodgkin's disease, unspecified type, intra-abdominal lymph nodes
20194	Hodgkin's disease, unspecified type, lymph nodes of axilla and upper limb
20195	Hodgkin's disease, unspecified type, lymph nodes of inguinal region and lower limb
20196	Hodgkin's disease, unspecified type, intrapelvic lymph nodes
20197	Hodgkin's disease, unspecified type, spleen
20198	Hodgkin's disease, unspecified type, lymph nodes of multiple sites
20200	Nodular lymphoma, unspecified site, extranodal and solid organ sites
20201	Nodular lymphoma, lymph nodes of head, face, and neck
20202	Nodular lymphoma, intrathoracic lymph nodes
20203	Nodular lymphoma, intra-abdominal lymph nodes
20204	Nodular lymphoma, lymph nodes of axilla and upper limb
20205	Nodular lymphoma, lymph nodes of inguinal region and lower limb

20206	Nodular lymphoma, intrapelvic lymph nodes
20207	Nodular lymphoma, spleen
20208	Nodular lymphoma, lymph nodes of multiple sites
20210	Mycosis fungoides, unspecified site, extranodal and solid organ sites
20211	Mycosis fungoides, lymph nodes of head, face, and neck
20212	Mycosis fungoides, intrathoracic lymph nodes
20213	Mycosis fungoides, intra-abdominal lymph nodes
20214	Mycosis fungoides, lymph nodes of axilla and upper limb
20215	Mycosis fungoides, lymph nodes of inguinal region and lower limb
20216	Mycosis fungoides, intrapelvic lymph nodes
20217	Mycosis fungoides, spleen
20218	Mycosis fungoides, lymph nodes of multiple sites
20220	Sezary's disease, unspecified site, extranodal and solid organ sites
20221	Sezary's disease, lymph nodes of head, face, and neck
20222	Sezary's disease, intrathoracic lymph nodes
20223	Sezary's disease, intra-abdominal lymph nodes
20224	Sezary's disease, lymph nodes of axilla and upper limb
20225	Sezary's disease, lymph nodes of inguinal region and lower limb
20226	Sezary's disease, intrapelvic lymph nodes
20227	Sezary's disease, spleen
20228	Sezary's disease, lymph nodes of multiple sites
20230	Malignant histiocytosis, unspecified site, extranodal and solid organ sites
20231	Malignant histiocytosis, lymph nodes of head, face, and neck
20232	Malignant histiocytosis, intrathoracic lymph nodes
20233	Malignant histiocytosis, intra-abdominal lymph nodes
20234	Malignant histiocytosis, lymph nodes of axilla and upper limb
20235	Malignant histiocytosis, lymph nodes of inguinal region and lower limb
20236	Malignant histiocytosis, intrapelvic lymph nodes
20237	Malignant histiocytosis, spleen
20238	Malignant histiocytosis, lymph nodes of multiple sites
20240	Leukemic reticuloendotheliosis, unspecified site, extranodal and solid organ sites
20241	Leukemic reticuloendotheliosis, lymph nodes of head, face, and neck
20242	Leukemic reticuloendotheliosis, intrathoracic lymph nodes
20243	Leukemic reticuloendotheliosis, intra-abdominal lymph nodes
20244	Leukemic reticuloendotheliosis, lymph nodes of axilla and upper arm
20245	Leukemic reticuloendotheliosis, lymph nodes of inguinal region and lower limb
20246	Leukemic reticuloendotheliosis, intrapelvic lymph nodes
20247	Leukemic reticuloendotheliosis, spleen
20248	Leukemic reticuloendotheliosis, lymph nodes of multiple sites
20250	Letterer-siwe disease, unspecified site, extranodal and solid organ sites
20251	Letterer-siwe disease, lymph nodes of head, face, and neck
20252	Letterer-siwe disease, intrathoracic lymph nodes
20253	Letterer-siwe disease, intra-abdominal lymph nodes
20254	Letterer-siwe disease, lymph nodes of axilla and upper limb
20255	Letterer-siwe disease, lymph nodes of inguinal region and lower limb
20256	Letterer-siwe disease, intrapelvic lymph nodes
20257	Letterer-siwe disease, spleen
20258	Letterer-siwe disease, lymph nodes of multiple sites
20260	Malignant mast cell tumors, unspecified site, extranodal and solid organ sites
20261	Malignant mast cell tumors, lymph nodes of head, face, and neck
20262	Malignant mast cell tumors, intrathoracic lymph nodes
20263	Malignant mast cell tumors, intra-abdominal lymph nodes
20264	Malignant mast cell tumors, lymph nodes of axilla and upper limb
20265	Malignant mast cell tumors, lymph nodes of inguinal region and lower limb
20266	Malignant mast cell tumors, intrapelvic lymph nodes
20267	Malignant mast cell tumors, spleen
20268	Malignant mast cell tumors, lymph nodes of multiple sites
20270	Peripheral T cell lymphoma, unspecified site, extranodal and solid organ sites
20271	Peripheral T cell lymphoma, lymph nodes of head, face, and neck
20272	Peripheral T cell lymphoma, intrathoracic lymph nodes
20273	Peripheral T cell lymphoma, intra-abdominal lymph nodes
20274	Peripheral T cell lymphoma, lymph nodes of axilla and upper limb
20275	Peripheral T cell lymphoma, lymph nodes of inguinal region and lower limb
20276	Peripheral T cell lymphoma, intrapelvic lymph nodes
20277	Peripheral T cell lymphoma, spleen
20278	Peripheral T cell lymphoma, lymph nodes of multiple sites
20280	Other malignant lymphomas, unspecified site, extranodal and solid organ sites

20281	Other malignant lymphomas, lymph nodes of head, face, and neck
20282	Other malignant lymphomas, intrathoracic lymph nodes
20283	Other malignant lymphomas, intra-abdominal lymph nodes
20284	Other malignant lymphomas, lymph nodes of axilla and upper limb
20285	Other malignant lymphomas, lymph nodes of inguinal region and lower limb
20286	Other malignant lymphomas, intrapelvic lymph nodes
20287	Other malignant lymphomas, spleen
20288	Other malignant lymphomas, lymph nodes of multiple sites
20290	Other and unspecified malignant neoplasms of lymphoid and histiocytic tissue, unspecified site, extranodal and solid organ sites
20291	Other and unspecified malignant neoplasms of lymphoid and histiocytic tissue, lymph nodes of head, face, and neck
20292	Other and unspecified malignant neoplasms of lymphoid and histiocytic tissue, intrathoracic lymph nodes
20293	Other and unspecified malignant neoplasms of lymphoid and histiocytic tissue, intra-abdominal lymph nodes
20294	Other and unspecified malignant neoplasms of lymphoid and histiocytic tissue, lymph nodes of axilla and upper limb
20295	Other and unspecified malignant neoplasms of lymphoid and histiocytic tissue, lymph nodes of inguinal region and lower limb
20296	Other and unspecified malignant neoplasms of lymphoid and histiocytic tissue, intrapelvic lymph nodes
20297	Other and unspecified malignant neoplasms of lymphoid and histiocytic tissue, spleen
20298	Other and unspecified malignant neoplasms of lymphoid and histiocytic tissue, lymph nodes of multiple sites
20300	Multiple myeloma, without mention of having achieved remission
20301	Multiple myeloma, in remission
20302	Multiple myeloma, in relapse
20310	Plasma cell leukemia, without mention of having achieved remission
20311	Plasma cell leukemia, in remission
20312	Plasma cell leukemia, in relapse
20380	Other immunoproliferative neoplasms, without mention of having achieved remission
20381	Other immunoproliferative neoplasms, in remission
20382	Other immunoproliferative neoplasms, in relapse
20400	Acute lymphoid leukemia, without mention of having achieved remission
20401	Acute lymphoid leukemia, in remission
20402	Acute lymphoid leukemia, in relapse
20410	Chronic lymphoid leukemia, without mention of having achieved remission
20411	Chronic lymphoid leukemia, in remission
20412	Chronic lymphoid leukemia, in relapse
20420	Subacute lymphoid leukemia, without mention of having achieved remission
20421	Subacute lymphoid leukemia, in remission
20422	Subacute lymphoid leukemia, in relapse
20480	Other lymphoid leukemia, without mention of having achieved remission
20481	Other lymphoid leukemia, in remission
20482	Other lymphoid leukemia, in relapse
20490	Unspecified lymphoid leukemia, without mention of having achieved remission
20491	Unspecified lymphoid leukemia, in remission
20492	Unspecified lymphoid leukemia, in relapse
20500	Acute myeloid leukemia, without mention of having achieved remission
20501	Acute myeloid leukemia, in remission
20502	Acute myeloid leukemia, in relapse
20510	Chronic myeloid leukemia, without mention of having achieved remission
20511	Chronic myeloid leukemia, in remission
20512	Chronic myeloid leukemia, in relapse
20520	Subacute myeloid leukemia, without mention of having achieved remission
20521	Subacute myeloid leukemia,in remission
20522	Subacute myeloid leukemia, in relapse
20530	Myeloid sarcoma, without mention of having achieved remission
20531	Myeloid sarcoma, in remission
20532	Myeloid sarcoma, in relapse
20580	Other myeloid leukemia, without mention of having achieved remission
20581	Other myeloid leukemia, in remission
20582	Other myeloid leukemia, in relapse
20590	Unspecified myeloid leukemia, without mention of having achieved remission
20591	Unspecified myeloid leukemia, in remission
20592	Unspecified myeloid leukemia, in relapse
20600	Acute monocytic leukemia, without mention of having achieved remission
20601	Acute monocytic leukemia,in remission
20602	Acute monocytic leukemia, in relapse
20610	Chronic monocytic leukemia, without mention of having achieved remission
20611	Chronic monocytic leukemia, in remission
20612	Chronic monocytic leukemia, in relapse

20620	Subacute monocytic leukemia, without mention of having achieved remission
20621	Subacute monocytic leukemia, in remission
20622	Subacute monocytic leukemia, in relapse
20680	Other monocytic leukemia, without mention of having achieved remission
20681	Other monocytic leukemia, in remission
20682	Other monocytic leukemia, in relapse
20690	Unspecified monocytic leukemia, without mention of having achieved remission
20691	Unspecified monocytic leukemia, in remission
20692	Unspecified monocytic leukemia, in relapse
20700	Acute erythremia and erythroleukemia, without mention of having achieved remission
20701	Acute erythremia and erythroleukemia, in remission
20702	Acute erythremia and erythroleukemia, in relapse
20710	Chronic erythremia, without mention of having achieved remission
20711	Chronic erythremia, in remission
20712	Chronic erythremia, in relapse
20720	Megakaryocytic leukemia, without mention of having achieved remission
20721	Megakaryocytic leukemia, in remission
20722	Megakaryocytic leukemia, in relapse
20780	Other specified leukemia, without mention of having achieved remission
20781	Other specified leukemia, in remission
20782	Other specified leukemia, in relapse
20800	Acute leukemia of unspecified cell type, without mention of having achieved remission
20801	Acute leukemia of unspecified cell type, in remission
20802	Acute leukemia of unspecified cell type, in relapse
20810	Chronic leukemia of unspecified cell type, without mention of having achieved remission
20811	Chronic leukemia of unspecified cell type, in remission
20812	Chronic leukemia of unspecified cell type, in relapse
20820	Subacute leukemia of unspecified cell type, without mention of having achieved remission
20821	Subacute leukemia of unspecified cell type, in remission
20822	Subacute leukemia of unspecified cell type, in relapse
20880	Other leukemia of unspecified cell type, without mention of having achieved remission
20881	Other leukemia of unspecified cell type, in remission
20882	Other leukemia of unspecified cell type, in relapse
20890	Unspecified leukemia, without mention of having achieved remission
20891	Unspecified leukemia, in remission
20892	Unspecified leukemia, in relapse
20900	Malignant carcinoid tumor of the small intestine, unspecified portion
20901	Malignant carcinoid tumor of the duodenum
20902	Malignant carcinoid tumor of the jejunum
20903	Malignant carcinoid tumor of the ileum
20910	Malignant carcinoid tumor of the large intestine, unspecified portion
20911	Malignant carcinoid tumor of the appendix
20912	Malignant carcinoid tumor of the cecum
20913	Malignant carcinoid tumor of the ascending colon
20914	Malignant carcinoid tumor of the transverse colon
20915	Malignant carcinoid tumor of the descending colon
20916	Malignant carcinoid tumor of the sigmoid colon
20917	Malignant carcinoid tumor of the rectum
20920	Malignant carcinoid tumor of unknown primary site
20921	Malignant carcinoid tumor of the bronchus and lung
20922	Malignant carcinoid tumor of the thymus
20923	Malignant carcinoid tumor of the stomach
20924	Malignant carcinoid tumor of the kidney
20925	Malignant carcinoid tumor of foregut, not otherwise specified
20926	Malignant carcinoid tumor of midgut, not otherwise specified
20927	Malignant carcinoid tumor of hindgut, not otherwise specified
20929	Malignant carcinoid tumor of other sites
20930	Malignant poorly differentiated neuroendocrine carcinoma, any site
20931	Merkel cell carcinoma of the face
20932	Merkel cell carcinoma of the scalp and neck
20933	Merkel cell carcinoma of the upper limb
20934	Merkel cell carcinoma of the lower limb
20935	Merkel cell carcinoma of the trunk
20936	Merkel cell carcinoma of other sites
20970	Secondary neuroendocrine tumor, unspecified site
20971	Secondary neuroendocrine tumor of distant lymph nodes
20972	Secondary neuroendocrine tumor of liver

20973	Secondary neuroendocrine tumor of bone
20974	Secondary neuroendocrine tumor of peritoneum
20975	Secondary Merkel cell carcinoma
20979	Secondary neuroendocrine tumor of other sites
2300	Carcinoma in situ of lip, oral cavity, and pharynx
2301	Carcinoma in situ of esophagus
2302	Carcinoma in situ of stomach
2303	Carcinoma in situ of colon
2304	Carcinoma in situ of rectum
2305	Carcinoma in situ of anal canal
2306	Carcinoma in situ of anus, unspecified
2307	Carcinoma in situ of other and unspecified parts of intestine
2308	Carcinoma in situ of liver and biliary system
2309	Carcinoma in situ of other and unspecified digestive organs
2310	Carcinoma in situ of larynx
2311	Carcinoma in situ of trachea
2312	Carcinoma in situ of bronchus and lung
2318	Carcinoma in situ of other specified parts of respiratory system
2319	Carcinoma in situ of respiratory system, part unspecified
2330	Carcinoma in situ of breast
2332	Carcinoma in situ of other and unspecified parts of uterus
23330	Carcinoma in situ, unspecified female genital organ
23331	Carcinoma in situ, vagina
23332	Carcinoma in situ, vulva
23339	Carcinoma in situ, other female genital organ
2334	Carcinoma in situ of prostate
2335	Carcinoma in situ of penis
2336	Carcinoma in situ of other and unspecified male genital organs
2337	Carcinoma in situ of bladder
2339	Carcinoma in situ of other and unspecified urinary organs
2340	Carcinoma in situ of eye
2348	Carcinoma in situ of other specified sites
2349	Carcinoma in situ, site unspecified
23770	Neurofibromatosis, unspecified
23771	Neurofibromatosis, type 1 [von recklinghausen's disease]
23772	Neurofibromatosis, type 2 [acoustic neurofibromatosis]
23773	Schwannomatosis
23779	Other neurofibromatosis
25801	Multiple endocrine neoplasia [MEN] type I
25802	Multiple endocrine neoplasia [MEN] type IIA
25803	Multiple endocrine neoplasia [MEN] type IIB
78951	Malignant ascites

Severe intellectual disability

Code	Description
317	Mild intellectual disabilities
3180	Moderate intellectual disabilities
3181	Severe intellectual disabilities
3182	Profound intellectual disabilities
319	Unspecified intellectual disabilities

Psychiatric chronic disease

Code	Description
2910	Alcohol withdrawal delirium
29181	Alcohol withdrawal
29500	Simple type schizophrenia, unspecified
29501	Simple type schizophrenia, subchronic
29502	Simple type schizophrenia, chronic
29503	Simple type schizophrenia, subchronic with acute exacerbation
29504	Simple type schizophrenia, chronic with acute exacerbation
29505	Simple type schizophrenia, in remission

29510	Disorganized type schizophrenia, unspecified
29511	Disorganized type schizophrenia, subchronic
29512	Disorganized type schizophrenia, chronic
29513	Disorganized type schizophrenia, subchronic with acute exacerbation
29514	Disorganized type schizophrenia, chronic with acute exacerbation
29515	Disorganized type schizophrenia, in remission
29520	Catatonic type schizophrenia, unspecified
29521	Catatonic type schizophrenia, subchronic
29522	Catatonic type schizophrenia, chronic
29523	Catatonic type schizophrenia, subchronic with acute exacerbation
29524	Catatonic type schizophrenia, chronic with acute exacerbation
29525	Catatonic type schizophrenia, in remission
29530	Paranoid type schizophrenia, unspecified
29531	Paranoid type schizophrenia, subchronic
29532	Paranoid type schizophrenia, chronic
29533	Paranoid type schizophrenia, subchronic with acute exacerbation
29534	Paranoid type schizophrenia, chronic with acute exacerbation
29535	Paranoid type schizophrenia, in remission
29550	Latent schizophrenia, unspecified
29551	Latent schizophrenia, subchronic
29552	Latent schizophrenia, chronic
29553	Latent schizophrenia, subchronic with acute exacerbation
29554	Latent schizophrenia, chronic with acute exacerbation
29555	Latent schizophrenia, in remission
29560	Schizophrenic disorders, residual type, unspecified
29561	Schizophrenic disorders, residual type, subchronic
29562	Schizophrenic disorders, residual type, chronic
29563	Schizophrenic disorders, residual type, subchronic with acute exacerbation
29564	Schizophrenic disorders, residual type, chronic with acute exacerbation
29565	Schizophrenic disorders, residual type, in remission
29570	Schizoaffective disorder, unspecified
29571	Schizoaffective disorder, subchronic
29572	Schizoaffective disorder, chronic
29573	Schizoaffective disorder, subchronic with acute exacerbation
29574	Schizoaffective disorder, chronic with acute exacerbation
29575	Schizoaffective disorder, in remission
29580	Other specified types of schizophrenia, unspecified
29581	Other specified types of schizophrenia, subchronic
29582	Other specified types of schizophrenia, chronic
29583	Other specified types of schizophrenia, subchronic with acute exacerbation
29584	Other specified types of schizophrenia, chronic with acute exacerbation
29585	Other specified types of schizophrenia, in remission
29590	Unspecified schizophrenia, unspecified
29591	Unspecified schizophrenia, subchronic
29592	Unspecified schizophrenia, chronic
29593	Unspecified schizophrenia, subchronic with acute exacerbation
29594	Unspecified schizophrenia, chronic with acute exacerbation
29595	Unspecified schizophrenia, in remission
29630	Major depressive affective disorder, recurrent episode, unspecified
29631	Major depressive affective disorder, recurrent episode, mild
29632	Major depressive affective disorder, recurrent episode, moderate
29633	Major depressive affective disorder, recurrent episode, severe, without mention of psychotic behavior
29634	Major depressive affective disorder, recurrent episode, severe, specified as with psychotic behavior
29635	Major depressive affective disorder, recurrent episode, in partial or unspecified remission
29636	Major depressive affective disorder, recurrent episode, in full remission
29640	Bipolar I disorder, most recent episode (or current) manic, unspecified
29641	Bipolar I disorder, most recent episode (or current) manic, mild
29642	Bipolar I disorder, most recent episode (or current) manic, moderate
29643	Bipolar I disorder, most recent episode (or current) manic, severe, without mention of psychotic behavior
29644	Bipolar I disorder, most recent episode (or current) manic, severe, specified as with psychotic behavior
29645	Bipolar I disorder, most recent episode (or current) manic, in partial or unspecified remission
29646	Bipolar I disorder, most recent episode (or current) manic, in full remission
29650	Bipolar I disorder, most recent episode (or current) depressed, unspecified
29651	Bipolar I disorder, most recent episode (or current) depressed, mild
29652	Bipolar I disorder, most recent episode (or current) depressed, moderate
29653	Bipolar I disorder, most recent episode (or current) depressed, severe, without mention of psychotic behavior
29654	Bipolar I disorder, most recent episode (or current) depressed, severe, specified as with psychotic behavior

29655	Bipolar I disorder, most recent episode (or current) depressed, in partial or unspecified remission
29656	Bipolar I disorder, most recent episode (or current) depressed, in full remission
29660	Bipolar I disorder, most recent episode (or current) mixed, unspecified
29661	Bipolar I disorder, most recent episode (or current) mixed, mild
29662	Bipolar I disorder, most recent episode (or current) mixed, moderate
29663	Bipolar I disorder, most recent episode (or current) mixed, severe, without mention of psychotic behavior
29664	Bipolar I disorder, most recent episode (or current) mixed, severe, specified as with psychotic behavior
29665	Bipolar I disorder, most recent episode (or current) mixed, in partial or unspecified remission
29666	Bipolar I disorder, most recent episode (or current) mixed, in full remission
2967	Bipolar I disorder, most recent episode (or current) unspecified
29680	Bipolar disorder, unspecified
29681	Atypical manic disorder
29682	Atypical depressive disorder
29689	Other bipolar disorders
2971	Delusional disorder
2973	Shared psychotic disorder
2979	Unspecified paranoid state
2989	Unspecified psychosis
29900	Autistic disorder, current or active state
29901	Autistic disorder, residual state
29910	Childhood disintegrative disorder, current or active state
29911	Childhood disintegrative disorder, residual state
29980	Other specified pervasive developmental disorders, current or active state
29981	Other specified pervasive developmental disorders, residual state
29990	Unspecified pervasive developmental disorder, current or active state
29991	Unspecified pervasive developmental disorder, residual state
30021	Agoraphobia with panic disorder
3003	Obsessive-compulsive disorders
30122	Schizotypal personality disorder
30300	Acute alcoholic intoxication in alcoholism, unspecified
30301	Acute alcoholic intoxication in alcoholism, continuous
30302	Acute alcoholic intoxication in alcoholism, episodic
30303	Acute alcoholic intoxication in alcoholism, in remission
30390	Other and unspecified alcohol dependence, unspecified
30391	Other and unspecified alcohol dependence, continuous
30392	Other and unspecified alcohol dependence, episodic
30393	Other and unspecified alcohol dependence, in remission
30420	Cocaine dependence, unspecified
30421	Cocaine dependence, continuous
30422	Cocaine dependence, episodic
30423	Cocaine dependence, in remission
30400	Opioid type dependence, unspecified
30401	Opioid type dependence, continuous
30402	Opioid type dependence, episodic
30403	Opioid type dependence, in remission
30470	Combinations of opioid type drug with any other drug dependence, unspecified
30471	Combinations of opioid type drug with any other drug dependence, continuous
30472	Combinations of opioid type drug with any other drug dependence, episodic
30473	Combinations of opioid type drug with any other drug dependence, in remission
3071	Anorexia nervosa
30751	Bulimia nervosa
